# Distinguishable DNA methylation defines a cardiac-specific epigenetic clock

**DOI:** 10.1186/s13148-023-01467-z

**Published:** 2023-03-29

**Authors:** A. Mongelli, S. Panunzi, M. Nesta, M. Gottardi Zamperla, S. Atlante, V. Barbi, V. Mongiardini, F. Ferraro, S. De Martino, L. Cis, A. Re, S. Maltese, T. Bachetti, M. T. La Rovere, F. Martelli, M. Pesce, S. Nanni, M. Massetti, A. Pontecorvi, A. Farsetti, C. Gaetano

**Affiliations:** 1 Laboratorio di Epigenetica, Istituti Clinici Scientifici (ICS) Maugeri IRCCS, 27100 Pavia, Italy; 2 National Research Council (CNR)-IASI, 00185 Rome, Italy; 3 Fondazione Policlinico Universitario A. Gemelli IRCCS, 00168 Rome, Italy; 4 Università Cattolica del Sacro Cuore, 00168 Rome, Italy; 5 National Research Council (CNR)-IRIB, 90146 Palermo, Italy; 6 Direzione Scientifica Centrale ICS Maugeri IRCCS, Pavia, Italy; 7 Dipartimento di Cardiologia ICS Maugeri and Direzione Scientifica ICS Maugeri Montescano IRCCS, Pavia, Italy; 8 Molecular Cardiology Laboratory, IRCCS Policlinico San Donato, San Donato Milanese, Milan Italy; 9 Unità di Ingegneria Tissutale Cardiovascolare, Centro Cardiologico Monzino IRCCS, 20138 Milan, Italy; 10 Present Address: Center for Translational and Experimental Cardiology (CTEC), University of Zurich, 8952 Schlieren, Switzerland; 11 Present Address: Molecular Medicine, Istituto Italiano di Tecnologia, Genoa, Italy

**Keywords:** Epigenetic clock, Aging, DNAmAge, Pyrosequencing, Cardiovascular disease, DNA methylation, DNAmAge, Heart biological age, Risk factors

## Abstract

**Background:**

The present study investigates whether epigenetic differences emerge in the heart of patients undergoing cardiac surgery for an aortic valvular replacement (AVR) or coronary artery bypass graft (CABG). An algorithm is also established to determine how the pathophysiological condition might influence the human biological cardiac age.

**Results:**

Blood samples and cardiac auricles were collected from patients who underwent cardiac procedures: 94 AVR and 289 CABG. The CpGs from three independent blood-derived biological clocks were selected to design a new blood- and the first cardiac-specific clocks. Specifically, 31 CpGs from six age-related genes, ELOVL2, EDARADD, ITGA2B, ASPA, PDE4C, and FHL2, were used to construct the tissue-tailored clocks. The best-fitting variables were combined to define new cardiac- and blood-tailored clocks validated through neural network analysis and elastic regression. In addition, telomere length (TL) was measured by qPCR. These new methods revealed a similarity between chronological and biological age in the blood and heart; the average TL was significantly higher in the heart than in the blood. In addition, the cardiac clock discriminated well between AVR and CABG and was sensitive to cardiovascular risk factors such as obesity and smoking. Moreover, the cardiac-specific clock identified an AVR patient's subgroup whose accelerated bioage correlated with the altered ventricular parameters, including left ventricular diastolic and systolic volume.

**Conclusion:**

This study reports on applying a method to evaluate the cardiac biological age revealing epigenetic features that separate subgroups of AVR and CABG.

**Supplementary Information:**

The online version contains supplementary material available at 10.1186/s13148-023-01467-z.

## Background

An increasing number of studies have demonstrated that epigenetics is influential in the occurrence and development of cardiac hypertrophy [1] and coronary artery disease (CAD) [2]. Specifically, DNA methylation signatures have been associated with various cardiac diseases. Moreover, it has been suggested that changes in this modification may predict future recurrence or complication [3]. However, whether, in the presence of specific pathophysiological conditions, the epigenetic landscape will impact the cardiac aging process is currently unclear.

The myocardium is a highly structured tissue consisting of different cell types, including cardiomyocytes, endothelial cells, fibroblasts, smooth muscle cells, inflammatory and microvascular cells, and a small pool of pluripotent stem cells [4]. Despite its heterogeneity, the cardiac muscle has intrinsic biological and molecular features that distinguish it from other tissues or organs. For example, unlike the bone marrow stem cell reservoir, which is virtually unlimited [5], the heart has a minimal regenerative capacity [6, 7]. Specifically, in a steady-state condition, the rate of heart renewal decreases annually from 1% at the age of 25 to 0.45% at 75, resulting in inefficient self-renewal [6]. In addition, a recent study comparing the cellular mass daily turnover of different human cell types reported that the cardiomyocytes’ rate approximates 0.001 g/day, lymphocytes rate is around 1 g/day, while erythrocytes and neutrophils’ rate is around 10 g/day [8]. Consistently, the adult cardiac muscle undergoes little changes in DNA methylation compared to its dynamic at the early embryonic and perinatal stages [9–11]. In addition, in rodents, the cardiac muscle seems to accumulate methylated cytosine forms, a feature that has also been associated with its meager endogenous proliferation rate [12, 13].

Variations in the level and distribution of methylated CpGs in the human genome and those of several animal species have been widely used to determine the biological age compared to the chronological one. Indeed, multiple epigenetic clocks based on genomic DNA CpG methylation patterns have been developed [14–18]. The most popular procedures for DNAmAge determination are based on evaluating many CpGs. They have been validated on cohorts of hundreds or thousands of blood-derived DNA samples [15, 19–21]. Their application contributed to defining the so-called DNA methylation age (DNAmAge), which in some cases may unravel the presence of a biological age deceleration or acceleration compared to the chronological one. An accelerated DNAmAge has been associated with the risk of developing diseases or a reduced healthspan and lifespan [22]. Multi-tissue epigenetic clocks have also been developed, allowing the simultaneous determination of DNAmAge in different organs of the same individual, including the heart [14, 23].

In parallel, other methods have been developed in humans and mice based on evaluating a reduced number of methylated CpGs measurable in whole blood DNA [16–18, 24–28]. Although applicable to multi-organ samples [27], these clocks seem to perform better on DNA obtained from the blood since the reduced number of CpGs limits their application to a multi-tissue evaluation. Nevertheless, these approaches are adequate to determine the individual DNAmAge [29]. Furthermore, they are technically based on the direct sequencing of selected CpGs, having a practical advantage in solving forensic problems or providing a rapid solution to processing many clinical or experimental samples at a negligible cost. Hence, although several multi-tissue methods for biological clock determination are available based on many CpGs common to different organs, the development of tissue-specific procedures based on a limited number of CpGs is still of interest to rapidly investigate unprecedented questions possibly influenced by aging-related organ-specific features [30].

The population of interest in this work comprises patients undergoing cardiac surgery for aortic valve replacement (AVR) or coronary artery bypass graft (CABG) whose auricle specimens and blood samples were made available to us. The DNAmAge differences between blood and cardiac tissues of the same individual were evaluated by comparing and combining three known and validated epigenetic clocks based on a reduced number of CpGs [16, 17, 24]. These methods were further developed to obtain novel tissue-specific algorithms based on different CpGs in the same amplicon of the pre-defined genes. The novel clocks were defined as the Mongelli & Panunzi (M&P) cardiac and blood models.

The study suggests that the heart may have intrinsic features that influence the organ-specific aging process defined as DNAmAge and identifies a group of AVR patients characterized by the worst ventricular parameters as significantly accelerated and distinguishable from the other participants.

## Results

### Definition of a blood-specific epigenetic clock

**Fig. 1:**
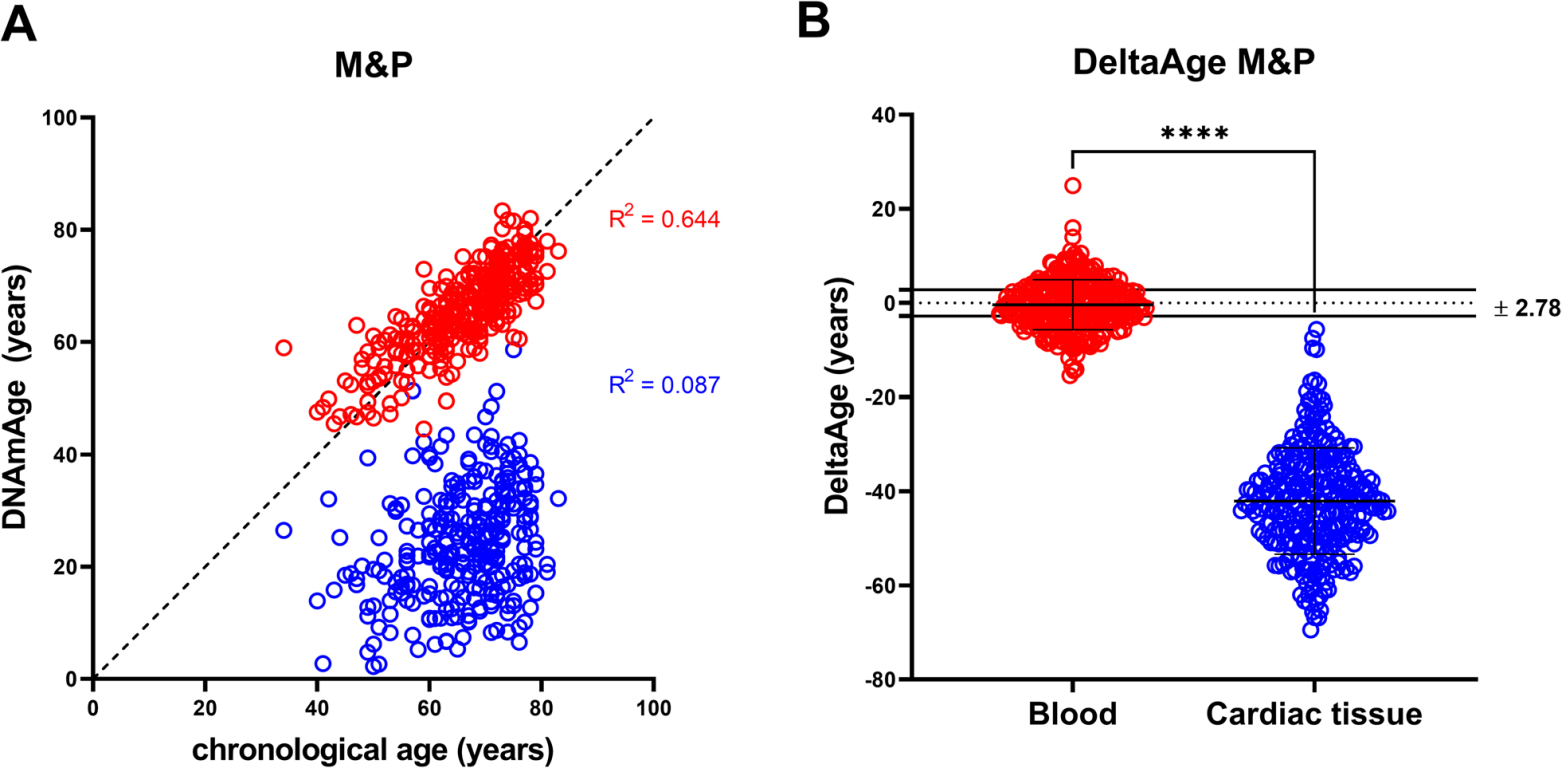
Mongelli&Panunzi (M&P) blood model. Red dots: blood samples (*n* = 313); blue dots: cardiac tissue samples (*n* = 313). **A** The correlation of chronological age and DNAmAge. Red dots align with the bisector, while blue dots do not. Chronological age of the sample (mean ± SD) 66.5 ± 9.7 years; M&P blood-specific formula on blood samples age: 65.7 ± 7.6 years (paired *T* test of chronological age vs. blood DNAmAge *p* = ns); M&P blood-specific on cardiac tissue samples: 24.0 ± 10.0 years (paired *t* test chronological age vs. cardiac tissue DNAmAge *p* value < 0.0001). **B** DeltaAges of blood and cardiac tissue. Blood – 0.40 ± 5.30 years; cardiac tissue − 42.0 ± 11.30. paired *T* test: *p* < 0.0001

Published blood-based pyrosequencing methods to evaluate the human biological age have been applied to our blood and auricle samples from the same individual (*n *= 383) (Additional file 1: Fig. S1A, C, E). As a result, in all epigenetic clocks [15, 16, 23], the heart was younger than the blood (Additional file 1: Fig. S1B, D, F). However, considering the intrinsic features of the heart, we reasoned that cardiac and blood-specific formulas could better represent the differences between the two tissues. Hence, we analyzed all CpGs (31 in total) present in the genes used to define the three blood-specific clocks, specifically Weidner [16], Zbiec-Piekarska [24], and Bekaert [17] (Additional file 1: Fig. S1 and Additional file 2: Table S1. These CpGs are distributed in the promoter region of the six genes of the above blood-specific clock models. We used these 31 CpGs to define our two clocks, named blood-specific M&P, described in this section, and cardiac-specific M&P epigenetic clock (see below paragraph 4.2). The Additional file 3: Fig. S2 depicts the heat map related to the univariable (Additional file 3: Fig. S2A) and multivariable (Additional file 3: Fig. S2B) procedures (Stepwise and Lasso regression, Recursive Feature Selection) employed in fitting CpGs for the blood samples of the training dataset (Additional file 2: Table S1). The blood-specific M&P model included only those predictors significant at a *p* level of 0.10: ASPA (CpG1); EDARADD (CpG2); ELOVL2 (CpG2, CpG3, CpG4, CpG5, and CpG6); FHL2 (CpG2, CpG3, CpG4, CpG5, and CpG10); ITGA2B (CpG2) and PDE4C (CpG5) (Additional file 4: Table S2). Figure 1 shows the blood-specific clock performance on the whole dataset. Additional file 2: Table S1 reports the correlation between the selected CpGs and the chronological age in the training and validation sample. Although some CpGs entered the model only with a linear term, some exhibit significant linear and quadratic coefficients. In addition, the chronological age in blood samples has been compared to that predicted by the M&P method, and no differences emerged (paired *t* test *p* value > 0.05). The complete blood-based formula is reported in Table 1.

**Table 1:** M&P epigenetic clock formulas

M&P epigenetic clocks	Formula
Blood	+ 44.6227153511 − 0.3019982767*EDARADD(CpG2) + 0.2328338368*ELOVL2(CpG2) − 0.5749152672*ELOVL2(CpG3) − 0.9899312404*ELOVL2(CpG6) + 0.6160217020*FHL2(CpG10) − 0.9602220285*FHL2(CpG2) + 2.4305557761*FHL2(CpG3) − 0.1896789356*FHL2(CpG4) − 0.0865888452*ITGA2B(CpG2) + 1.0970579110*PDE4C(CpG5) − 0.0006750886*ASPA(CpG1)^2^ + 0.0025856173*ELOVL2(CpG4)^2^ + 0.0111994179*ELOVL2(CpG6)^2^ − 0.0131271779*FHL2(CpG10)^2^ + 0.0095023800*FHL2(CpG2)^2^ − 0.0185023682*FHL2 (CpG3)^2^ − 0.0102953607*PDE4C (CpG5)^2^
Heart	+ 70.773560858 + 0.182565872*EDARADD(CpG1) + 0.848763871*ELOVL2(CpG2) + 0.100544271*ELOVL2 (CpG5) − 1.531702946*ELOVL2(CpG7) + 1.000713439*FHL2(CpG1) − 0.674486107*FHL2(CpG4) + 1.170946852*FHL2(CpG5) + 0.816922873*FHL2(CpG7) − 0.641544097*FHL2(CpG8) − 0.958506956*ITGA2B(CpG1) + 0.211886446*ITGA2B(CpG2) − 0.008128604*ELOVL2(CpG3)^2^ + 0.016263489*ELOVL2(CpG7)^2^ − 0.026586745*FHL2(CpG5)^2^ + 0.008172081*ITGA2B(CpG1)^2^ − 0.026087696*PDE4C (CpG3)^2^ + 0.014023034*PDE4C(CpG5)^2^

To determine DNAmAge, coefficients must be multiplied by the percentage of CpG methylation

The median absolute deviation (MAD) of the DeltaAge represents the range of normality. In this work, the MAD value obtained from the Mongelli & Panunzi (M&P) method is 2.78 (Fig. 1B) compared to Bekaert’s 3.34; Weidner’s 9.55, and Zbiec-Piekarska 7.08 (Additional file 1: Fig. S1, panels B, D, F). Interestingly, the blood-based M&P clock applied to the cardiac samples showed a significant deceleration of the DNAmAge paralleled by a DeltaAge reduction (Fig. 1A, B, blue dots). This evidence prompted us to explore the possibility of developing a cardiac-specific clock. Additional file 4: Table S2 reports the list of CpGs used to define the blood and cardiac epigenetic clocks and their localization on the genome (human genome version Hg38).

The performance of all models used to define DNAmAge and DeltaAge values from the blood samples in the training and testing datasets are reported in Additional file 5: Fig. S3 and Additional file 6: Fig. S4, respectively. In addition, Additional file 7: Fig. S5 compares the different DeltaAge results obtained after applying the different DNAmAge algorithms; Additional file 8: Table S3 reports the performance of each method in correctly predicting the chronological age, whereas Additional file 9: Table S4 shows the correlation between chronological age and DNAmAge in each dataset. Additionally, M&P blood-specific algorithm has been applied to blood samples of healthy volunteers (Additional file 10: Fig. S6). The average of their chronological ages is 57.4 years with a standard deviation of 4.8, while the DNAmAge is 56.1 ± 5.2. Additional file 10: Fig. S6A shows the scatter plot between chronological age and DNAmAge in healthy volunteers. The paired *t* test between Chronological and DNAmAge reveals no significant difference (*p* value = 0.097). Moreover, 57.7% of the sample is distributed within the MAD value of ± 2.78, meaning that the DNAmAge is predominantly aligned to the chronological one (Additional file 10: Fig. S6B).

### Definition of a cardiac-specific epigenetic clock

**Fig. 2:**
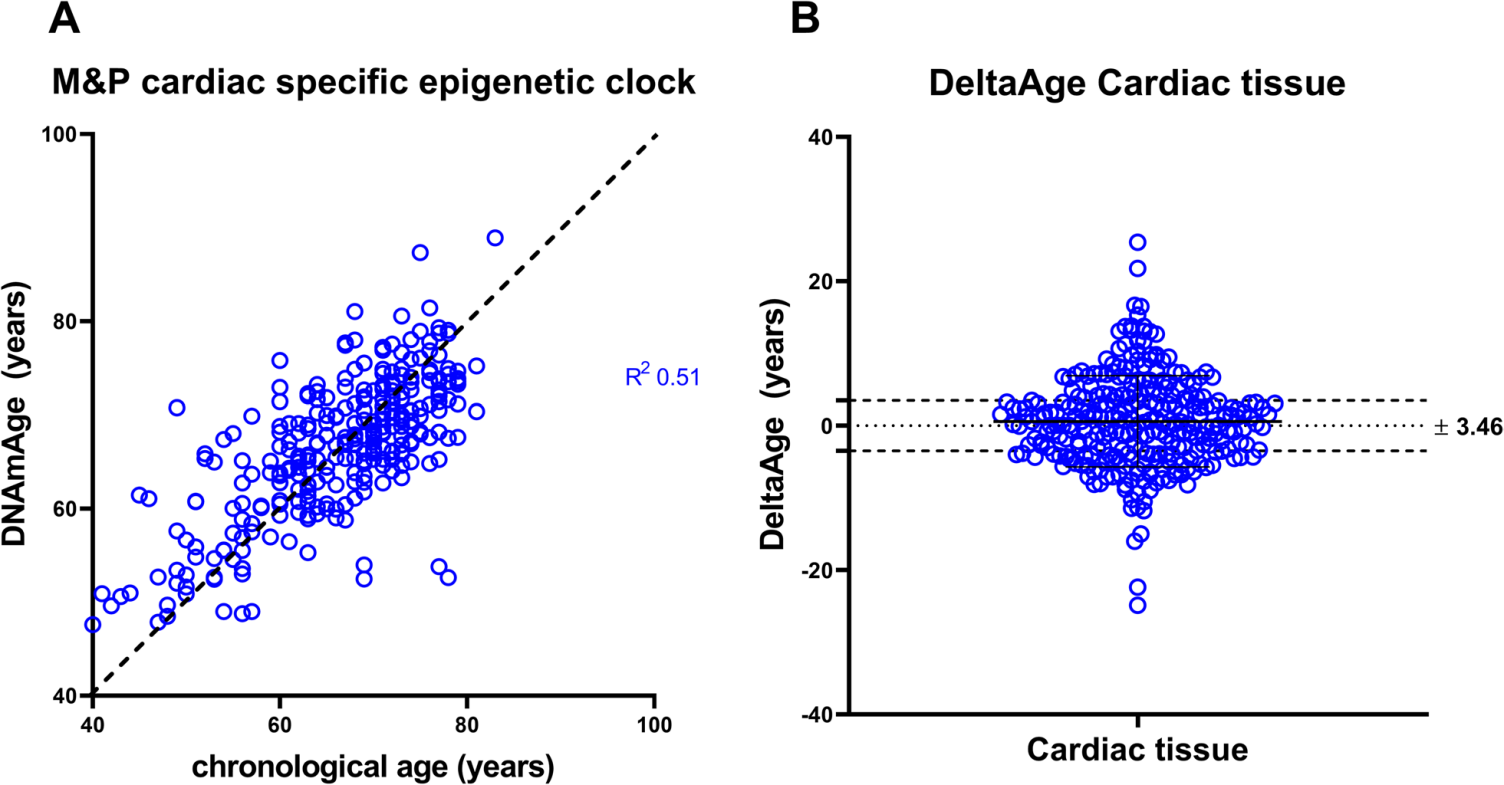
M&P cardiac-specific formula applied to cardiac tissue. **A** Comparison of chronological age vs. DNAmAge. Samples align to the bisector. Chronological age versus cardiac-specific DNAmAge (mean ± SD, 66.4 ± 7.4). paired *T* test *p* value = ns. **B** DeltaAge values were obtained after applying the cardiac-specific formula (0.60 ± 6.3; MAD 3.46)

Attempting to evaluate the DNAmAge of the heart, we found that different CpGs of the same genes showed to be significant predictors of the donors' chronological age (Additional file 11: Fig. S7). The heat maps in Additional file 11: Fig. S7 report the univariable relationships (Additional file 11: Fig. S7A) and the multivariable associations (Additional file 11: Fig. S7B) from the three selection procedures of the considered CpGs. The final cardiac model included the following CpGs as predictors of chronological age: EDARADD (CpG1), ELOVL2 (CpG2, CpG3, CpG5, and CpG7), FHL2 (CpG1, CpG4, CpG5, CpG7, and CpG8), ITGA2B (CpG1 and CpG2), PDE4C (CpG3 and CpG5) (Additional file 4: Table S2 and Table 1 for the cardiac-specific formula). Also, in this case, some CpGs entered the model only linearly, others with a first and a second-order relationship. Finally, applying the new formulation to the samples of cardiac origin, we obtained a sample alignment with an R^2^ of 0.510 (Fig. 2A), and the fitting of the chronological age of the donors produced a MAD of 3.46 (Fig. 2B).

**Fig. 3:**
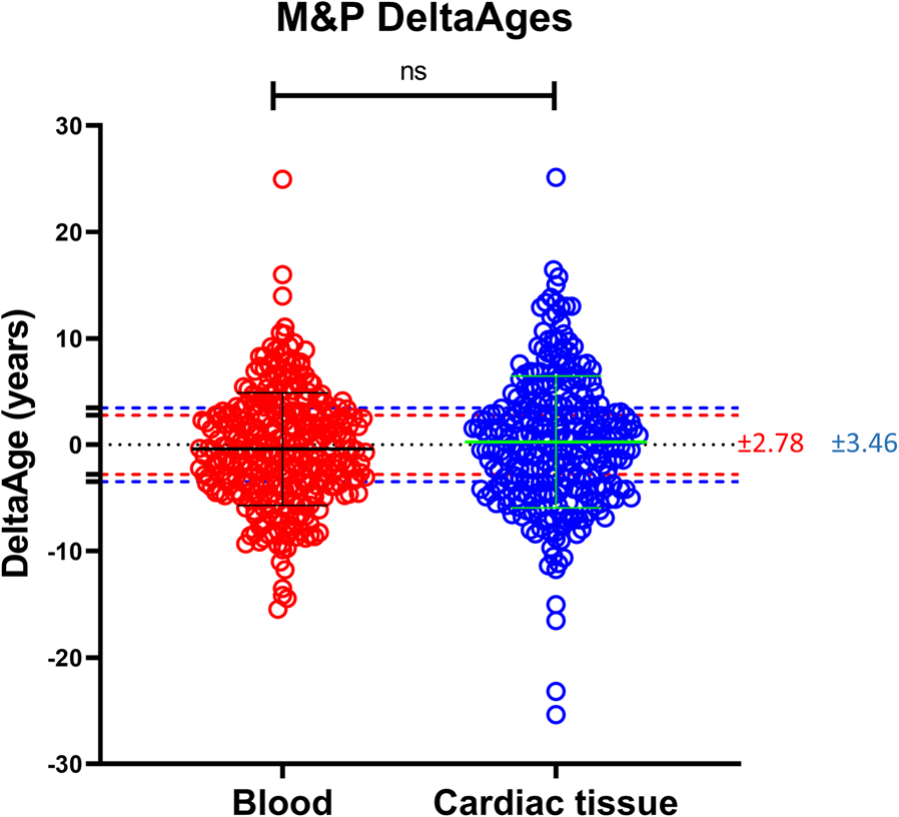
DeltaAges compares blood and cardiac tissue using a specific M&P algorithm for both samples. Red lines delimit the blood MAD (± 2.78), while blue lines delimit the cardiac tissue MAD (± 3.46). Paired *t* test reveals a non-significative (ns) *p* value (> 0.05)

In addition, no differences in DeltaAges were observed between the training and testing groups (Additional file 12: Fig. S8A–C). The M&P blood and cardiac-specific clocks were then applied to all samples, and no differences emerged between the blood and cardiac DeltaAge, suggesting that the cardiac tissue might not be younger than the blood and that, on average, both tissues reflected the chronological age of the donor (Fig. 3).

Additional file 13: Table S5 shows the results of the correlation between CpG methylation levels and chronological age. The mean and the median of each epigenetic clock are reported in Additional file 14: Table S6. Finally, in Additional file 15: Table S7, the descriptive statistic of the M&P cardiac-specific model is reported.

Finally, neural networks (NNs) were built by considering all the CpGs of the studied genes as inputs. The 31 CpGs were entered as inputs for the biological age prediction. Panel A and B of Additional file 16: Fig. S9 show the neural networks associated with the blood and cardiac tissue models, respectively. Additional file 17: Table S8 reports the prediction abilities of the NNs in terms of absolute DeltaAge and MAD. A minimal improvement is observed with the machine learning approach when considering the cardiac tissue: MAD: 3.46 (M&P method and 14 CpGs from 5 genes) vs. 3.28 (NN, with 31 CpGs from six genes).

On the other hand, no improvement could be observed applying NN to the blood tissue: MAD: 2.78 (M&P method and 12 CpGs from 6 genes) vs. 3.83 from the NN. This result suggests that the proposed M&P blood-tailored clock, with only 12 CpGs from 6 genes, performs better than an approach that involves more complex nonlinear relationships and a more significant number of features. Therefore, the results prompted us to use the proposed M&P (more parsimonious) methods for the chronological age prediction from the blood and heart samples.

### DNAmAge distribution between AVR and CABG and associated risk factors

In this study, we investigated whether AVR or CABG influenced the global average distribution of DeltaAge in the blood and cardiac cohorts. The features of our population study are reported in Table 2.

**Table 2:** Population study’s features

Population study (tot *n* = 383)	AVR (*n* = 94)		CABG (*n* = 289)		Chi-squared *p* value overall
Male	58 (15.1%)		229 (59.8%)		**0.0007**
Female	36 (9.4%)		60 (15.7%)		
BMI ≥ 30	Male	11 (12.9%)	Male	54 (63.5%)	0.20
	Female	6 (7.1%)	Female	14 (16.5%)	
Smoker	Male	20 (13.8%)	Male	101 (69.7%)	**0.005**
	Female	10 (6.9%)	Female	14 (9.7%)	
Former smoker	Male	0	Male	35 (85.4%)	n.a
	Female	0	Female	6 (14.6%)	
Congestive heart failure	Male	5 (33.3%)	Male	1 (6.7%)	0.31
	Female	8 (53.3%)	Female	1 (6.7%)	
Atrial fibrillation	Male	13 (29.5%)	Male	16 (36.4%)	0.11
	Female	11 (25.0%)	Female	4 (9.1%)	
Hypertension	Male	43 (13.2%)	Male	203 (62.3%)	**0.0004**
	Female	29 (8.9%)	Female	51 (15.6%)	
Dyslipidemia	Male	25 (9.1%)	Male	182 (66.4%)	** < 0.0001**
	Female	22 (8.0%)	Female	45 (16.4%)	

In bold are reported the significant *p* values

**Fig. 4:**
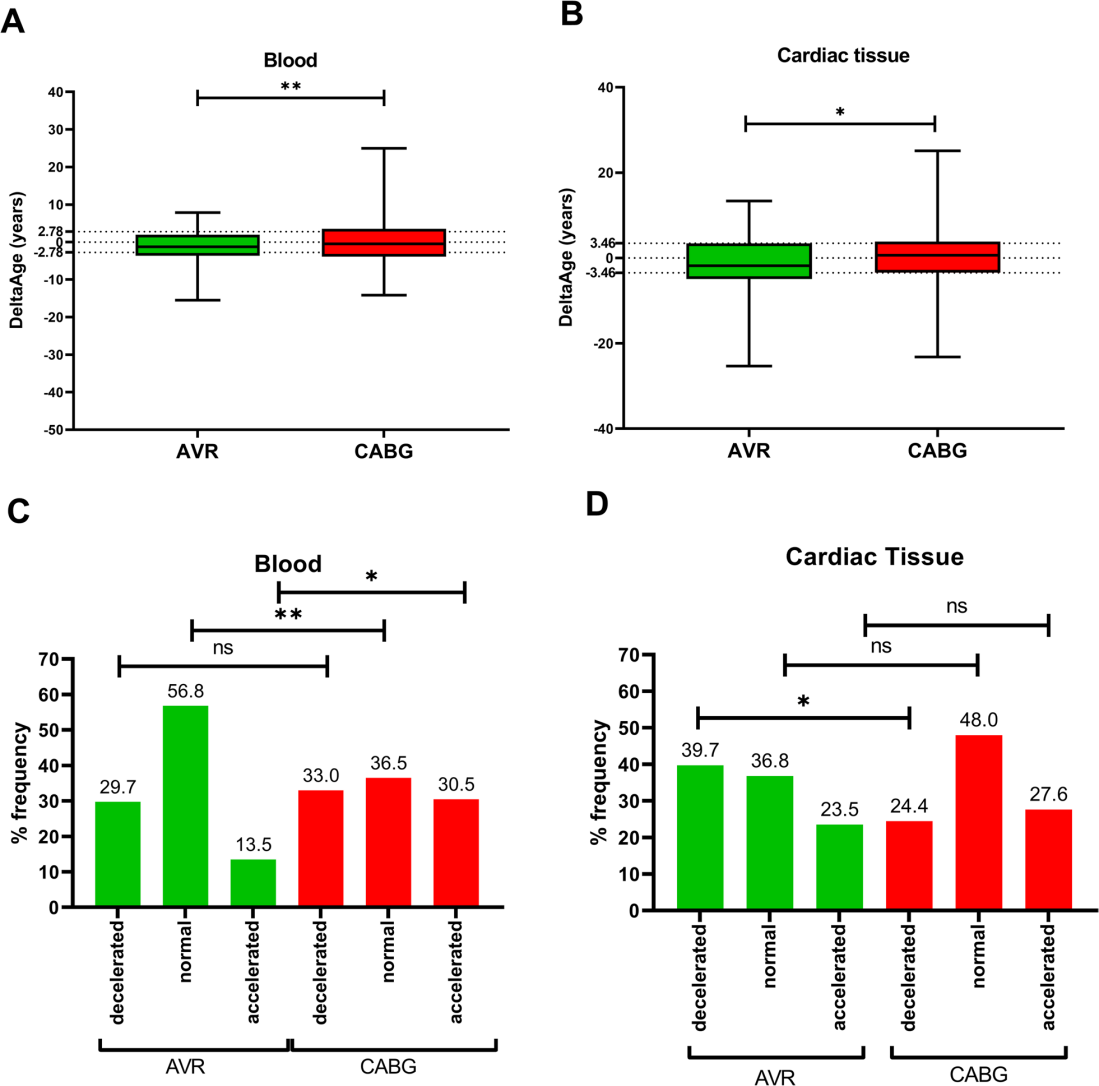
AVR and GABG DeltaAges. **A** M&P blood DeltaAge. Differences between AVR (*n* = 74) and CABG (*n* = 239) in blood DeltaAges (Overall Chi-squared test *p* value = 0.002). **B** M&P cardiac tissue DeltaAge. AVR (*n* = 68) patients reveal lower values of DeltaAge compared to CABG (*n* = 224) (Overall Chi-squared test *p* value = 0.049). **C** Blood sample size and percentage of DeltaAge distribution according to clinical classification. Differences between AVR(decelerated *n* = 22; normal = 42; and accelerated *n* = 10) and CABG AVR (decelerated *n* = 79; normal = 87; and accelerated *n* = 73) have been tested utilizing Post Hoc Analysis for Pearson’s Chi-Squared Test. Significant differences were found when comparing normal and accelerated groups (*p* value 0.006 and 0.011, respectively). **D** Cardiac tissue distribution has been analyzed utilizing Post Hoc Analysis for Pearson’s Chi-Squared Test. Significant differences were found in AVR versus CABG decelerated groups (*p* value 0.045). AVR distribution: decelerated *n* = 27; normal = 25; and accelerated *n* = 16; CABG distribution: decelerated *n* = 55; normal = 108; and accelerated *n* = 61

Average DeltaAges for blood were − 1.4 ± 4.8 and − 0.10 ± 5.4 in AVR and CABG, respectively (*p* = 0.049) (Fig. 4A). However, the average DeltaAge for cardiac samples in the AVR group was − 1.3 ± 6.9 vs. 0.7 ± 5.9 in the CABG group (*p* = 0.02) (Fig. 4B). Next, we investigated the detailed distribution of the biological age. Specifically, groups with decelerated, regular, and accelerated biological age compared with the chronological one were detected in the AVR vs. CABG blood and heart samples (Fig. 4C, D; *p* = 0.002). Differences emerged in the blood's normal and accelerated AVR vs. CABG groups, showing that 56.8% of AVRs had a normal DeltaAge distribution while 13.5% were accelerated compared to 36.5% and 30.5% of the CABGs (*p* = 0.006; *p* = 0.011 respectively). However, in the cardiac tissue, we found different percentages of normal/decelerated/accelerated in the two groups (*p* = 0.048), with 39.7% of the AVRs having a decelerated DeltaAge, indicating this condition is enriched among AVRs and in comparison with 24.5% for the CAGB (Fig. 4D).

Finally, we explored whether crucial CVD risk factors influenced the distribution of AVR and CABG’s DeltaAge. The Cochran–Mantel–Haenszel test was used to assess if the distributions in Normal/Decelerated/Accelerated in the AVR and CABG groups were conditionally independent in each stratum of the CVD risk factors. The test was significant when considering BMI as a stratification factor (*p* = 0.002), with a stronger association in the group with BMI < 30 kg/m^2^. Post hoc analysis for Pearson’s Chi-Squared Test, with *p* values adjusted for multiple comparisons by the Benjamini–Hochberg correction, was used to test differences in percentages between AVR and CABG. In the group with BMI < 30 kg/m^2^, we found that the AVG group had about twice the percentage of normal and about half of the accelerated individuals compared to the CABG group (Table 2). When the DeltaAge distributions were compared between AVR and CABG in the two subsamples of individuals with and without smoke habits, the risk factor influenced the association: smoking history determined a more significant percentage of AVR donors with a normal DeltaAge. At the same time, CABGs were characterized by accelerated individuals (Table 3). On the other hand, no significant association was found for the heart samples, even if the AVR group presented more significant percentages of individuals with decelerated DNAmAge (Table 4).

**Table 3:** The different levels of blood epigenetic age distribution in the risk factors

Blood DeltaAge	AVR (*n* = 74)			CABG (*n* = 239)			Ratio AVR/CABG (*p* value)		
	Decelerated (%)	Normal (%)	Accelerated (%)	Decelerated (%)	Normal (%)	Accelerated (%)	Decelerated	Normal	Accelerated
BMI < 30 kg/m^2^	25.9	58.6	15.5	36.0	33.7	30.3	0.72 (0.47)	1.7 (0.002)**	0.5 (0.08)
BMI ≥ 30 kg/m^2^	43.7	50.0	6.3	25.0	43.7	31.25	1.7 (0.41)	1.14 (1.0)	0.20 (0.1)
Smoker	29.2	62.5	8.3	34.8	31.8	33.3	0.8 (1)	2 (0.01)*	0.25 (0.04)*
No smoker	30.0	54.0	16.0	30.6	41.7	27.8	0.98 (1.0)	1.3 (0.44)	0.6 (0.32)

Inside each risk factor level, the DeltaAge distribution was evaluated for possible differences between AVR and CABG. Notably, most AVR donors with BMI lower than 30 kg/m^2^ had normal DeltaAge. On the other hand, a significantly higher percentage of smoker CABG donors had accelerated DeltaAges. Differences between AVR and CABG have been tested utilizing post hoc analysis for Pearson’s chi-squared test with *p* values adjusted for multiple comparisons by the Benjamini–Hochberg *p* correction. Symbols * and ** indicate conditions reaching statistical significance

**Table 4:** The cardiac epigenetic age distribution in the risk factors’ different levels

Cardiac tissue DeltaAge	AVR (*n* = 68)			CABG (*n* = 224)			Ratio AVR/CABG (*p* value)		
	Decelerated (%)	Normal (%)	Accelerated (%)	Decelerated (%)	Normal (%)	Accelerated (%)	Decelerated	Normal	Accelerated
BMI < 30 kg/m^2^	40.7	35.2	24.1	25.3	51.8	22.9	1.6 (0.09)	0.7 (0.10)	1.0 (1.0)
BMI ≥ 30 kg/m^2^	35.7	42.9	21.4	22.4	37.9	39.7	1.6 (0.90)	1.1 (1.0)	0.54 (0.60)
Smoker	35.0	30.0	35.0	24.4	45.5	30.1	1.43 (0.94)	0.66 (0.58)	1.1 (1.0)
No smoker	41.67	39.5	18.7	25.0	51.0	24.0	1.7 (0.11)	0.78 (0.58)	0.78 (1.0)

Inside each risk factor level, the DeltaAge distribution was evaluated for possible differences between AVR and CABG. Differences between AVR and CABG have been tested using Post Hoc Analysis for Pearson’s Chi-Squared Test with *p* values adjusted for multiple comparisons by the Benjamini–Hochberg *p* correction. Even if not significant, the AVR group presented larger percentages of individuals with decelerated DNAmAge

The cardiological condition (AVR/CABG) was also tested in a multinomial model for the prediction of DeltaAges. In the blood sample, the risk of accelerated DeltaAges was more than three times greater in CABGs than in AVRs (OR: 3.4, *p* = 0.001), while in the cardiac samples, the risk of decelerated DeltaAges was reduced by more than half for the CABG group compared to AVRs (OR: 0.47, *p* = 0.02).

### Cardiac structural alteration prevails among accelerated AVRs

**Fig. 5:**
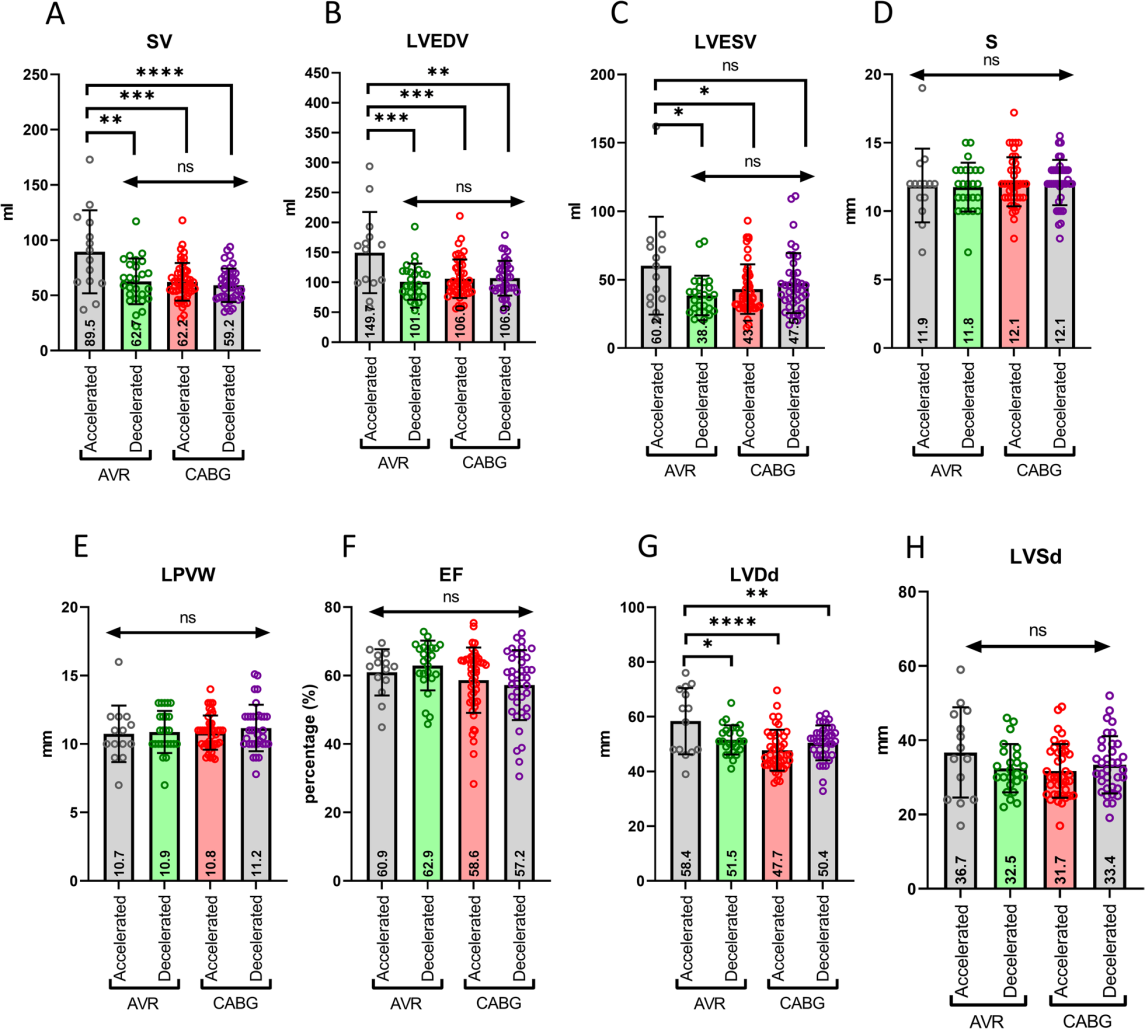
Echocardiography distinguishes accelerated and decelerated AVR and CABG. At the bottom of each bar, the average value is reported. **A** SV: Stroke volume. No differences between AVR decelerated and CABG groups. However, significant differences arose in AVR accelerated compared to the others. **B** LVEDV: left ventricular end-diastolic volume. No differences between decelerated AVR and the CABG groups. On the other hand, the accelerated AVR revealed significant differences compared to all others. **C** LVESV: left ventricle end-systolic volume, differences emerged in the accelerated AVR vs. decelerated AVR and versus accelerated CABG. No differences were detected evaluating S: septal; LPVW: left posterior ventricular wall thickness; EF: ejection fraction; and LVSd: left systolic ventricle diameter (panels **D**, **E**, **F**, and **H**, respectively). Interestingly, panel **G** shows that in the accelerated AVRs, the LVDd: left ventricle diastolic diameter is significantly higher than in the other groups (*p* < 0.05). *p < 0.05; **p < 0.01; ***p < 0.001; ****p < 0.0001

Seventy-seven percent of individuals with a BMI higher than 30 kg/m^2^ and with smoke habits were CABG, and smoke was significantly associated (*p* < 0.001) with the specific pathophysiological condition considering that 84% of smokers were in the CABG group. These results indicate that the reduced incidence of these risk factors in the AVRs might contribute to their predominantly normal or younger cardiac biological age. This evidence prompted us to evaluate whether structural and functional features of the heart could be associated with accelerated or decelerated AVRs compared to CABG. Therefore, we explored whether these groups had echocardiographic physical and functional parameters distributed differently. The following were evaluated: the stroke volume (SV), the left ventricular end-diastolic and systolic volumes (LVEDV; LVESV), the septal (S) and left posterior ventricular wall (LVPW) thickness, the ejection fraction (EF), and the diameter of the diastolic and systolic left ventricle (LVDd; LVSd). Interestingly, DeltaAge acceleration in the AVRs correlated with a significant increase in SV, LVEDV, and LVESV compared to the decelerated AVRs and both CABG groups (Fig. 5A–C).

Statistical analysis indicated that differences were significant for the SV values (*p* < 0.0001). In particular, significant variances emerged among: (i) accelerated AVR vs. decelerated AVR (*p* = 0.0007); (ii) accelerated AVR vs. accelerated CABG (*p* < 0.0001); and (iii) accelerated AVR vs. decelerated CABG (*p* < 0.0001) (Fig. 5A). A similar result emerged for LVEDV (Fig. 5B), LVESV (Fig. 5C), and LVDd (Fig. 5G).

## Discussion

In this study, we addressed the cardiac-specific biological age compared to that of the peripheral blood and whether the presence of specific pathophysiological conditions leading to major cardiac surgery influences this aspect. The details of our cohort study are reported in Table 1.

Since Hannum [15] and Horvath [14] works, multiple biological clocks have been developed by screening many CpGs with microarrays with or without pyrosequencing validation [14–17, 24]. Although multi-organs algorithms have already been described [14], they are almost exclusively based on analyzing DNA samples originating from peripheral blood. However, internal organs may have intrinsic features, such as different growth rates [8], specific telomere lengths [31], and organ-specific variation in the DNA methylation level [32] that might influence the epigenetic landscape. For example, despite its essential pumping function characterized by spontaneous electrical activity and contractility, the heart has the least regeneration capacity, with a self-renewal rate between 1 and 4% during the entire lifespan [6]. In addition, the average cardiac telomere length appears more extended than that of leukocytes or other internal organs, including the kidney and liver, see Additional file 18: Fig. S10 [33, 34]. This evidence could be associated with the heart's insufficient regenerative capacity, possibly reducing telomere attrition. In this context, it is also unknown what the cardiac stem cells’ contribution may be. Several cardiac precursors with regenerative properties have been reported in different cardiac regions, including the atria. Overall, they are recognized by some markers described as CD31^neg^/CD45^neg^/c-kit^pos^/Sca-1^pos^/Abcg-2^pos^/PDGF-Ralpha^pos^ (see references [4, 35] and bibliography therein).

Interestingly, since his seminal work, Horvath postulated that cardiac stem cells could influence the cardiac DNAmAge [14]. However, the contribution of resident cardiac stem cells to heart bioage remains unclear, particularly in pathophysiological conditions affecting heart function. Further investigation is required to elucidate this exciting aspect.

Lastly, in mice, the cardiac tissue seems prone to accumulate methylated cytosines, an epigenetic modification enrichment that has been proposed to be associated with reduced organ turnover [12, 13, 36]. On the opposite, the blood has a rapid turnover and exhibits a progressive shortening in telomere length that parallels the aging process and has been proposed as a valid parameter associated with the risk of cardiovascular accidents [37, 38]. Thus, in a physiological context, the cardiac muscle's intrinsic mechanical and metabolic features may impact the organ-specific aging process differently than in other organs.

Here, we propose two different pyrosequencing-based algorithms tailored to the cardiac and blood tissues and applicable to predicting the blood and cardiac DNAmAge in patients undergoing cardiac surgery. Their accuracy is in the range of several other algorithms, including those based on evaluating multiple CpGs, such as that initially described by Horvath [14]. Specifically, we used 31 CpGs present in 6 previously validated age-related genes [16–18, 24].

According to this approach, our analysis showed that distinct combinations of some of these CpGs were necessary to define the blood- and cardiac-specific methods to determine the individual biological age in the human blood and heart with a MAD of 2.78 and 3.46, respectively. Furthermore, applying the new methods to the blood and cardiac cohorts of samples showed a good correlation between chronological and predicted ages: *R*^2^ =  0.63 and 0.52, respectively. This approach determined an average biological age of 65.9 ± 7.1 for the blood and 65.9 ± 6.3 for the heart overlapping with the cohort's average chronological age of 66.5 ± 9.7. Consistently, in the blood samples from healthy controls, the *R*^2^ was 0.468 with an average chronological age of 57.4 ± 4.8, perfectly centered by the predicted age of 56.1 ± 5.2. We called these new methods as the M&P blood and cardiac clocks. The condition determining the CpG switch in the heart and blood is currently unknown; however, it might be associated with organ-specific metabolic conditions.

Our results disagree with previous observations reporting that the heart has a younger biological age than blood [14, 23, 39]. The explanation for the discrepancy might rely on i) the limited access to cardiac samples that might have reduced the accuracy of these previous analyses, ii) the algorithm used in previous works which were not tailored to the heart, or iii) on intrinsic cardiac features, including telomere length and a different DNA methylation enrichment [40]. Altogether these variables could reflect the presence of peculiar intrinsic cardiac features influencing the clock performance.

The new methods were developed using samples from donors undergoing cardiac surgery for AVR or CABG. In the blood, the M&P clock well predicted the biological age compared with the chronological within a MAD of 2.78, which is one of the lowest values reported among the methods based on a reduced number of CpGs [16–18, 24–26]. In addition, this algorithm discriminated between the two groups and correlated with specific risk factors (Tables 3, 4). Despite a similar chronological age, CABG patients had shorter telomeres than AVR (Additional file 18: Fig. S10), confirming the acceleration of the aging process in whole blood. Regarding the heart, we observed that samples from the AVR group had an average DeltaAge significantly lower than those with CABG (Fig. 4A). Furthermore, for the blood samples, CABGs presented a risk of exhibiting accelerated DeltaAges more than three times higher than AVRs (*p* = 0.011), while for the ischemic heart, the risk of falling into the decelerated DeltaAge group was significantly reduced by more than half compared to AVRs (*p* = 0.045) (Additional file 19: Table S9). This evidence suggests epigenetic alterations may accumulate in the ischemic heart, pushing toward an accelerated DNAmAge.

Investigating whether this distribution was associated with specific cardiovascular risk factors, it emerged that in the blood, a BMI < 30 kg/m^2^ characterized most AVR donors with normal or decelerated DNAmAge. In contrast, smoking history was associated with accelerated CABGs. Furthermore, most AVR individuals had normal or decelerated cardiac biological age compared to the CABG group. However, in our cohort, those AVR with an accelerated DNAmAge correlated well with a higher LVED, LVES, and stroke volume despite an ejection fraction falling within a range of normality as often reported for these patients (Fig. 5).

Understanding whether these findings could be associated with specific and intrinsic cardiac differences requires further investigation. However, a possibility to explain the differences emerging between AVR and CABG could be related to the different impact of mechanical cues on the cellular reprogramming occurring in the ischemic myocardium vs. that of valvular patients. In this regard, it is essential to highlight that the mechanical decompensation of the heart in valve disease is similar to cardiac hypertrophy due to pressure overload [41], while patients with CAD are generally prone to dilated cardiomyopathy. Since the two pathologic settings differ in the impact of the mechanical load on the structure of the myocardium and the myocardial cells [42], different epigenetic setups could be established in the two conditions [1, 2]. This hypothesis is supported by evidence showing that exposure of cells to mechanical cues undergoes specific epigenetic programming [43], possibly a consequence of mechanical effects [44] and that mechanical-dependent alterations in the nuclear shape affect chromatin organization with the acquisition of pathological phenotypes [45]. Interestingly, it has been recently reported that the YAP/TAZ signaling is involved in the negative regulation of cell senescence [46]. This observation supports the possibility that changes in cardiac mechanics could be at the basis of the DNAmAge acceleration observed in the AVR subpopulation.

This study has substantial limitations, including the low number of samples evaluated, the absence of a control group of age-matched healthy cardiac samples, and the relatively homogeneous distribution of the patient's chronological age limited to a restricted range. This situation reflects the objective difficulty of obtaining cardiac tissue from younger donors. Moreover, the absence of a follow-up did not allow for monitoring the evolution of the epigenetic modifications occurring after cardiac surgery. In addition, all the cardiac samples were obtained from the right atrium and might not reflect the epigenetic features of other organ districts, precisely that of the left ventricle, which is functionally the most critical part of the heart [47]. It is conceivable that many CpGs, such as those measurable with a methyl-chip hybridization, could provide additional information. Additional studies are necessary to address this crucial aspect. Nevertheless, our data provide unprecedented information about the cardiac biological age and indicate that no evident differences exist compared to peripheral blood [39]. Finally, evidence is provided that sensitive cardiac and blood-based algorithms realized with minimal CpGs are simple and suitable for virtually all laboratories, including those in a clinical setting.

Furthermore, our cardiac-specific algorithm allowed the identification of specific AVR and CABG subpopulations dictating indications for a more intense clinical follow-up. Moreover, the novel methods defined a cardiac-tailored epigenetic clock that shows how the heart's biological age is, on average, in line with the individual chronological one. Lastly, the cardiac clock detects a population of AVR patients biologically “younger” than CABGs [39] and a subgroup of accelerated AVRs presenting structural and functional cardiac alterations, possibly early signs of heart failure.

## Conclusions

In conclusion, we propose that the estimation of DNAmAge might be implemented in diagnostic procedures to personalize the treatment. Specifically, the application of this method to the cardiovascular field may affect prognosis, treatments, side effects, and mortality rates, significantly benefiting the patient from this information.

## Material and methods

### Patient enrolment

Three hundred eighty-three patients have been enrolled from 2019 to 2021 at the Department of Cardiovascular and Thoracic Sciences, Fondazione Policlinico Universitario A. Gemelli IRCCS-Università Cattolica, Rome, Italy (protocol number: 46406/18; ID: 2303, date of approval December 4, 2018), and informed consent was obtained from each patient. All procedures followed the principles expressed in the Declaration of Helsinki, the institutional regulation, and Italian laws and guidelines. Two hundred eighty-nine were coronary aortic bypass graft (CABG) surgery, and ninety-four were aortic valve replacement (AVR). Two hundred eighty-eight samples formed the training group to develop the new algorithms, and ninety-five were used as a testing group. Blood and auricles were obtained after signed informed consent from all participants.

### DNA extraction from whole blood

QIAmp DNA blood mini kit (Qiagen; cat. 51106) has been used to isolate genomic DNA from 200 ul of peripheral blood in EDTA in association with automated QIACube (Qiagen, cat. 9002160) according to manufacturer instructions.

### DNA extraction from cardiac auricle

Stainless Steel Beads 5 mm (Qiagen; cat. 69989) have been used to homogenize cardiac auricles (~ 25 mg). Tissue Lyzer (Qiagen; cat. 85600) has been set for 4 min at 40 Hz. To samples, 180 ul of ATL buffer (Qiagen, cat. 939011) and 20 ul of Proteinase K (Qiagen, cat. 91311) were added. Then, the samples were incubated at 56 °C for 10 min. The DNA extraction has been successfully automated by QIACube (Qiagen, cat. 9002160) according to manufacturer instructions.

### Bisulfite conversion

1 µg of DNA has been used for the conversion with Epitect fast DNA bisulfite (Qiagen, cat. 59824) following the manufacturer instructions associated with RotorGene 2plex HRM (Qiagen, cat. 9001560) and QIACube automated system. Then, 2ul of converted DNA was quantified with QIAxpert (Qiagen; cat. 9002340).

### Polymerase chain reactions

Following the manufacturer’s instructions, PCR reaction mixes have been performed using the PyroMark PCR kit (Qiagen, cat. 978103), and 50 ng of bisulfite-converted DNA has been used for the amplification. The PCR protocol was performed as follows: 95 °C for 15 min of initial denaturation; 95 °C for 30 s of denaturation; 56 °C for 30 s of annealing; 72 °C for 30 s of elongation and 72 °C for 10 min of final elongation. Denaturation, annealing, and elongation have been repeated 42 times.

PCR primers:

**Table:** 

Name	Modification 5′	Sequence (5′ → 3′)
fw_ELOVL2	BIOTIN	AGGGGAGTAGGGTAAGTGAGG
rv_ELOVL2		AACAAAACCATTTCCCCCTAATAT
fw_FHL2		TGTTTTTAGGGTTTTGGGAGTATAG
rv_FHL2	BIOTIN	ACACCTCCTAAAACTTCTCCAATCTCC
fw_ASPA		TGTTGAAGAATATATATAAAAGGTTGT
rv_ASPA	BIOTIN	ATCTTACCCAAAATTTTCAAAATCAAA
fw_ITGA2B		AGGAGTTTTGTTTTTAAGGGATTTAT
rv_ITGA2B	BIOTIN	AAACTCTTTAACCATTAAAACTTAA
fw_PDE4C		GTAGGAGGAAAAGGGTTAGGAGAG
rv_PDE4C	BIOTIN	CCCAAACCCCTTTCTCTAAC
fw_EDARADD		GGAGTTTGTTATGGAAGAAGTAATAG
rv_EDARADD	BIOTIN	ATCCTCCCACCTACAAATTC

### Pyrosequencing

The amplicons have been sequenced to check the methylation level in each CpG site. PyroMark Q24 Advanced Reagents (Qiagen, cat. 970902) have been loaded in the PyroMark Q24 Cartridge (Qiagen, cat. 979202) following the manufacturer instructions, and 5ul of PCR product has been added to the reaction mix.

Then, the samples were shacked at Room Temperature for 15 min at 1400 rpm.

Successively, the samples underwent the PyroMark Q24 Vacuum Station (Qiagen, cat. 9001515) procedure in which the target sequences were purified and put into an annealing buffer containing the sequencing primer [0,375 uM].

The sequence 5′ → 3′ of sequencing primers are:

- seq_ELOVL2: ACAACCAATAAATATTCCTAAAACT
- seq_FHL2: GGTTTTGGGAGTATAGT
- seq_ASPA: TGAAGAATATATATAAAAGGTTGTT
- seq_ITGA2B: GGATTAAGAGTAAATAGTGTG
- seq_PDE4C: GAATAGAAGAGTTGTTGGATG
- seq_EDARADD: TGTTATGGAAGAAGTAATAGA

Then, the plate containing the sequence to analyze and the primer was heated at 80 °C for 5 min. Successively, the PyroMark Q24 Advanced (Qiagen, cat. 9001514) has been set to analyze the following sequences:

- ELOVL2: CCRTAAACRTTAAACCRCCRCRCRAAACCRAC
- FHL2: AGTTATYGGGAGYGTYGTTTTYGGYGTGGGTTTTYGGGYGYGAGTTTYGGAYG
- AGGTTTGGGYGYGG
- ASPA: ATTTTTGGAGGAATTTATGGGAATGAGTTAATYGGAGTATTTTTGGTTAAGTAT
- TGG TTAGAGAATGGYGTTGAGAT
- ITGA2B: TTTAATGTTGTGTTTAYGTGTGTTAGTTTAYGYGGTTAGTTTGAGGAGTTAGG
- PDE4C: YGGATGGGGYGTYGGGGTTGTYGTTATAGGTGTTTYGGGGTTTT
- EDARADD: TTGYGAGAAGATGTTYGTTGG

### Statistical methods

The whole dataset was randomly divided into a training and a testing sample. The testing dataset was built from a subsample presenting at least 90% observations and constituted 35% of the total subjects. For both the cardio and blood dataset, a multivariable linear model for chronological age prediction was built based on the methylation values of some gene sites. All the considered gene-site are reported in additional figures for the cardio and blood tissue, respectively, along with the associated *p* values from a univariable model. The two models were built by including a subset of variables chosen according to the results from three independent selection procedures: a stepwise regression (combining both forward and backward selection), the Lasso (Least Absolute Shrinkage and Selection Operator) regression, where the tuning parameter lambda was set to the optimum value from a 30-cross-validation procedure, and the recursive feature elimination (RFE) algorithm which tries all possible solutions (up to the maximum number of possible features). RFE method also uses a repeated 30-cross-validation approach with ten repeats to improve the performance of feature selection. Finally, all the procedures were applied to a multivariable model, including only the predictors, resulting in significance at a *p* level of 10% in the univariable models. Variables were tested both in their linear and quadratic form. Only predictors that resulted significantly at 10% in at least one procedure entered the final multivariable model. The selected model removed the predictors, which in the final multivariable model exhibited a *p* value greater than 0.10. The two models derived by the above procedure have been called M&P models. Heatmaps were used to summarize the results: cell colors represent the entity of the regression coefficients associated with each predictor; values inside the cells are the *p* values associated with the regression coefficients. The models' performance was evaluated by computing the deviations between chronological ages and predicted ages; means, medians, trimmed means, and dispersion measures such as standard and median absolute deviation (MAD) were reported. The new models (M&P) were compared with the Bekaert, Weidner, and Zbiec-Piekarska algorithms. All the comparisons were made by applying the three known algorithms only to the gene sites from the blood samples. The procedure described above, adopted for selecting the predictors to be inserted into the final model, was compared with the performance of a Neural Network (NN) for the regression approach. NNs are machine learning algorithms that help predict the response (dependent variable) from many inputs (independent variables). Two NNs were built to model the chronological age as dependent on the methylation values of the gene sites from the blood and cardiac tissue. For the NN construction, all the analyzed CpGs of all the considered genes were included as inputs. The number of hidden layers was tuned by using bootstrapping. All the analyses were conducted in R (R Core Team 2021. URL https://www.R-project.org/).

### Telomere length quantification

The chromosome end has been quantified by PCR Real Time of Absolute Human Telomere Length Quantification qPCR Assay Kit (ScienCell, cat. 8918) following the manufacturer's instructions. In addition, 2 ng of DNA has been used for telomere analysis. The DNA reference used for the kb estimation (lot. #30521) presents TL of 726 ± 70 kb per diploid cell.

## Supplementary Information

**Additional file 1. Figure S1.** Blood-specific epigenetic clocks estimated by pyrosequencing. Red dots: blood; blue dots: cardiac tissue. Both samples underwent the application of already-known blood-based epigenetic clock algorithms. **A** the estimation of biological age (DNAmAge) developed by Beakert et al., blood samples are aligned to the bisector, while the cardiac tissue samples are not. **B** The results of the subtraction of chronological age to biological age (DeltaAge) after applying Bekaert’s formula: blood (mean ± SD) − 0.29 ± 8.2 years and cardiac tissue − 28.23 ± 11.3 years. **C** Weidner et al. algorithm. Blood and cardiac tissue samples are not aligned with the bisector. **D** DeltaAge is calculated after Weidner's formula blood + 11.8 ± 13.8 years and cardiac tissue − 8.27 ± 16.6 years. **E** Zbiec-Piekarska et al. biological clock. **F** DeltaAges calculation after applying Zbiec-Piekarska’s DNAmAge algorithm blood − 8.97 ± 9.3 years and cardiac tissue − 31.8 ± 11.6 years. All paired *t* tests of blood, and cardiac tissue DeltaAge reveal a *p* value < 0.0001.

**Additional file 2. Table S1**. Correlation between chronological age and blood CpG Methylation levels in different datasets. Values in bold refer to significant correlations.

**Additional file 3. Figure S2.** Heat map summarizing analyses of blood samples CpGs. **A** Results for all CpG analyzed in a univariable model. **B** Results from the Lasso regression, Stepwise regression, and Recursive feature selection for final model identification. Cell color represents the entity of the regression coefficient b for each CpG predicting the increase/decrease in aging in a univariable (**A**) and multivariable (**B**) analysis. Cell value represents the *p* value associated with the respective coefficients b. Variable name starting with “Sq.” indicates that the variable enters the model in quadric form.

**Additional file 4. Table S2.** Gene target and CpG were analyzed to estimate tissue-specific epigenetic clocks.

**Additional file 5. Figure S3.** Blood models of epigenetic clocks in training group in blood and cardiac tissue samples. **A** M&P model; **B** Bekaert; **C** Weidner; **D** Zbiec-Piekarska.

**Additional file 6. Figure S4.** blood models of epigenetic clocks in the blood and cardiac tissue samples testing group. **A** M&P model; **B** Bekaert; **C** Weidner; **D** Zbiec-Piekarska.

**Additional file 7. Figure S5.** Comparison of blood DeltaAges. The value 2.78 refers to the MAD of the M&P blood-based epigenetic clock.

**Additional file 8. Table S3.** Blood models comparison in training, testing, and whole sample groups.

**Additional file 9. Table S4.** Correlation between epigenetic clocks and chronological age. The r and R2 of each epigenetic clock are reported.

**Additional file 10. Figure S6.** Blood of healthy control group (*n* = 26). **A** Correlation between chronological age (mean ± SD) 57.4 ± 4.8 and DNAmAge 56.1 ± 5.2 years estimated by M&P blood formula (paired *t* test > 0.05). **B** Blood DeltaAge of healthy controls mean ± SD (− 1.34 ± 4.0). 57.7% of DeltaAge falls within the range of normality (± 2.78); 30.8% of volunteers are decelerated, while 11.5% are accelerated.

**Additional file 11. Figure S7.** Heat map summarizing analyses of cardiac samples CpGs. **A** Results for all CpG analyzed in a univariable model. **B** Results from the Lasso regression, Stepwise regression, and Recursive feature selection for final model identification. Cell color represents the entity of the regression coefficient b for each CpG predicting the increase/decrease in aging in a univariable (**A**) and multivariable (**B**) analysis. Cell value represents the P value associated with the respective coefficients b. Variable name starting with “Sq.” indicates that the variable enters the model in the quadric form.

**Additional file 12. Figure S8.** M&P cardiac model in training and testing groups. **A** Chronological versus DNAmAge in the training group; **B** Chronological versus DNAmAge in the testing group. **C** comparison of training and testing DeltaAges. No differences between cohorts (*p* value: ns).

**Additional file 13. Table S5.** Correlation between chronological age and cardiac tissue CpG Methylation levels in the different datasets. Values in bold refer to significant correlations.

**Additional file 14. Table S6.** The mean and median of different datasets in different epigenetic clocks.

**Additional file 15. Table S7.** Descriptive statistics of M&P cardiac model in training and testing groups. *p* value training versus testing: ns.

**Additional file 16. Figure S9.** Diagrams of the Neural Networks employed for the blood and cardiac tissue. **A** Plot of the Neural Network for the chronological age prediction from blood CpGs samples. **B** Plot of the Neural Network for the chronological age prediction from cardiac CpGs samples. Each node represents an input CpG, while edges represent the weights between layers. The thickness of the edge is proportional to the magnitude of each weight. Positive weights are plotted as black lines; negative weights as grey lines. The Bias nodes cover the same role as an intercept in a regression model. I = input node, O =output node, H = hidden node, B = bias weight.

**Additional file 17. Table S8.** Performance of the NNs for blood and cardiac tissue in training, testing, and whole sample groups.

**Additional file 18. Figure S10.** Telomere length (TL) of blood and cardiac tissue of AVR and CABG patients. The telomere length (kb) has been estimated by qPCR. Blood AVR TL mean and SD amount at 2.61 ± 0.57 kb; CABG blood TL is 2.42 ± 0.84 kb. Welch’s *t* test reveals a *p* value of 0.026. On the other hand, no differences arise in cardiac tissue (Welch’s *t* test *p* = 0.49) in which AVR TL is 4.48 kb ± 1.24 and CABG 4.60 ± 1.77 kb.

**Additional file 19. Table S9.** Sample size and percentage of DeltaAge distribution according to clinical classification. Differences between AVR and CABG have been tested utilizing Post Hoc Analysis for Pearson’s Chi-Squared Test. In blood, significant differences were found when comparing regular and accelerated groups; in the heart, the difference arises in the decelerated groups. Overall: Chi-Squared test.

## Data Availability

The datasets used and/or analyzed during the current study are available from the corresponding author upon reasonable request.

## Abbreviations

ASPA: Aspartoacylase
AVR: Aortic valve replacement
CABG: Coronary artery bypass graft
CAD: Coronary artery disease
CpG: Cytosine-guanine dinucleotide
CVDs: Cardiovascular diseases
DeltaAge: DNAmAge—chronological age (years)
DNAmAge: DNA methylation age
EDARADD: Ectodysplasin-A receptor-associated adapter protein
EF: Ejection fraction
ELOVL2: Fatty acid elongase 2
FHL2: Four and A half LIM domains 2
ITGA2B: Integrin alpha-IIb
LVDd: Left ventricle diastolic diameter
LVEDV: Left ventricle end-diastolic volume
LVESV: Left ventricle end-systolic volume
LVSd: Left ventricle systolic diameter
M&P: Mongelli and Panunzi
MAD: Mean absolute deviation
NN: Neuronal network
PDE4C: Phosphodiesterase 4C
S: Septum
SV: Stroke volume
