# Supplementary figures and images for: Distinguishable DNA methylation defines a cardiac-specific epigenetic clock
Source: Clin Epigenetics. 2023 Mar 29;15:53. doi: 10.1186/s13148-023-01467-z (PMC10053964; doi:10.1186/s13148-023-01467-z)

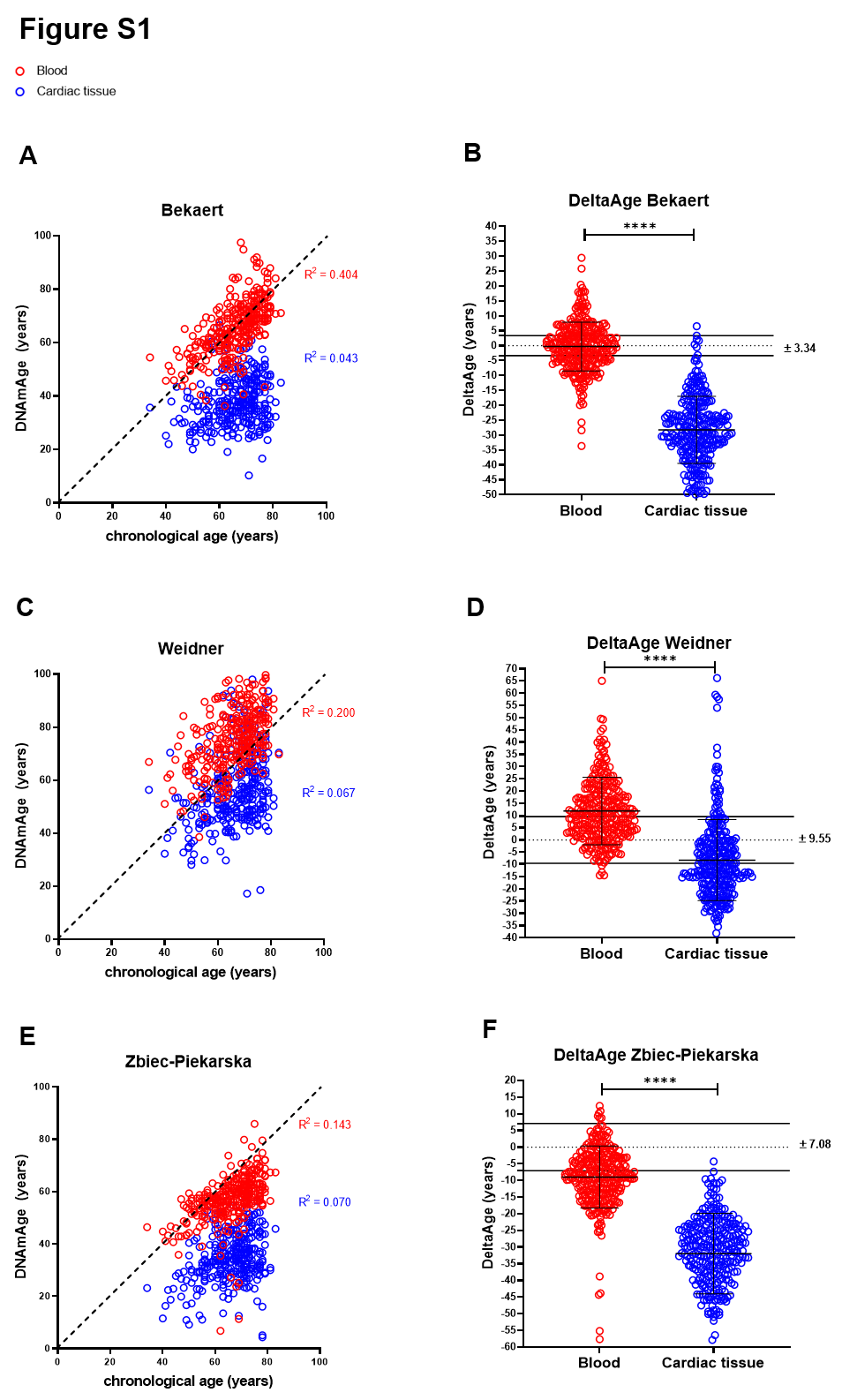

Supplement: Supplementary file 1 — Additional file 1. Figure S1. Blood-specific epigenetic clocks estimated by pyrosequencing. Red dots: blood; blue dots: cardiac tissue. Both samples underwent the application of already-known blood-based epigenetic clock algorithms. A the estimation of biological age (DNAmAge) developed by Beakert et al., blood samples are aligned to the bisector, while the cardiac tissue samples are not. B The results of the subtraction of chronological age to biological age (DeltaAge) after applying Bekaert’s formula: blood (mean ± SD) − 0.29 ± 8.2 years and cardiac tissue − 28.23 ± 11.3 years. C Weidner et al. algorithm. Blood and cardiac tissue samples are not aligned with the bisector. D DeltaAge is calculated after Weidner's formula blood + 11.8 ± 13.8 years and cardiac tissue − 8.27 ± 16.6 years. E Zbiec-Piekarska et al. biological clock. F DeltaAges calculation after applying Zbiec-Piekarska’s DNAmAge algorithm blood − 8.97 ± 9.3 years and cardiac tissue − 31.8 ± 11.6 years. All paired t tests of blood, and cardiac tissue DeltaAge reveal a p value < 0.0001. [file 13148_2023_1467_MOESM1_ESM.tif]

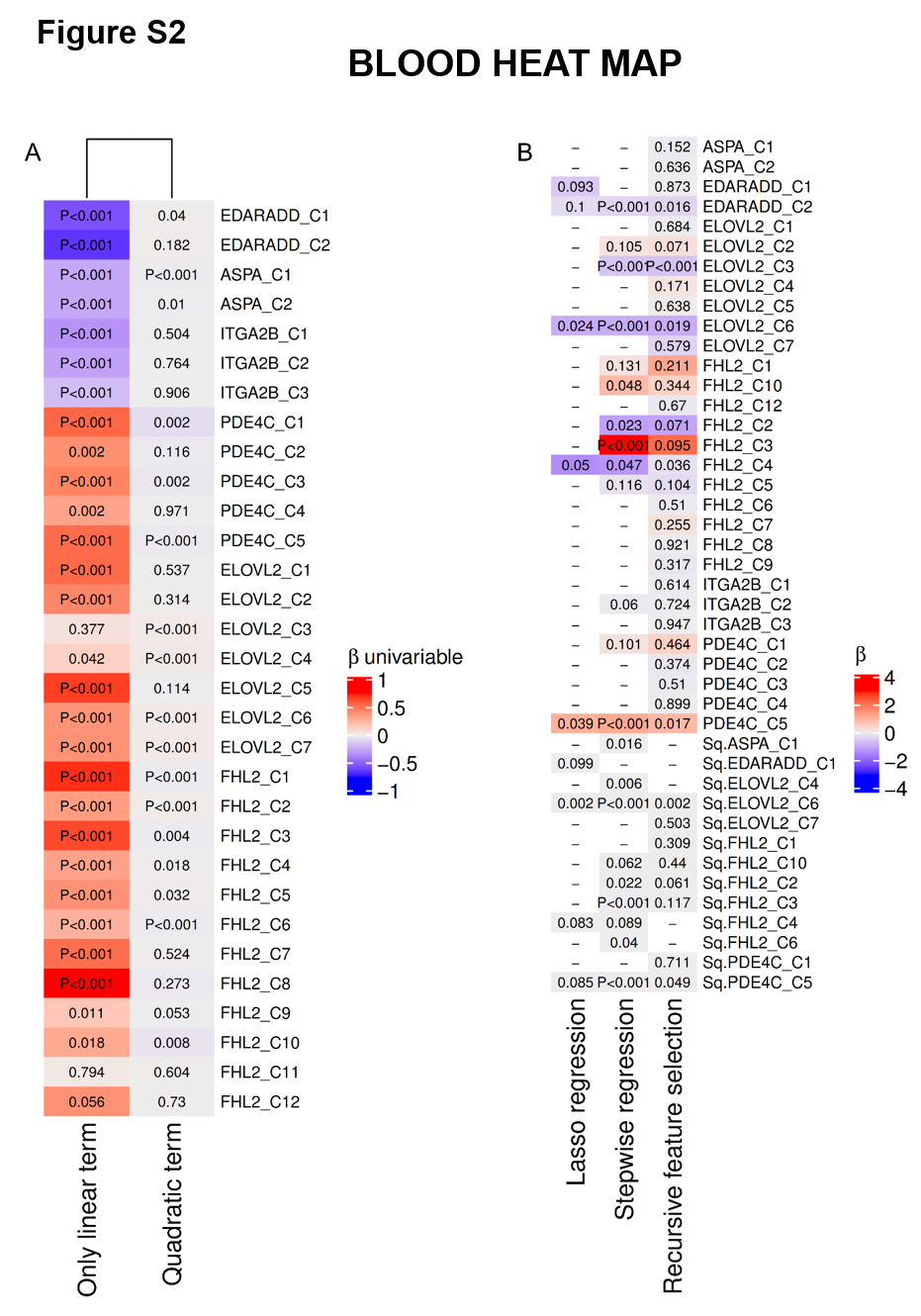

Supplement: Supplementary file 3 — Additional file 3. Figure S2. Heat map summarizing analyses of blood samples CpGs. A Results for all CpG analyzed in a univariable model. B Results from the Lasso regression, Stepwise regression, and Recursive feature selection for final model identification. Cell color represents the entity of the regression coefficient b for each CpG predicting the increase/decrease in aging in a univariable (A) and multivariable (B) analysis. Cell value represents the p value associated with the respective coefficients b. Variable name starting with “Sq.” indicates that the variable enters the model in quadric form. [file 13148_2023_1467_MOESM3_ESM.tif]

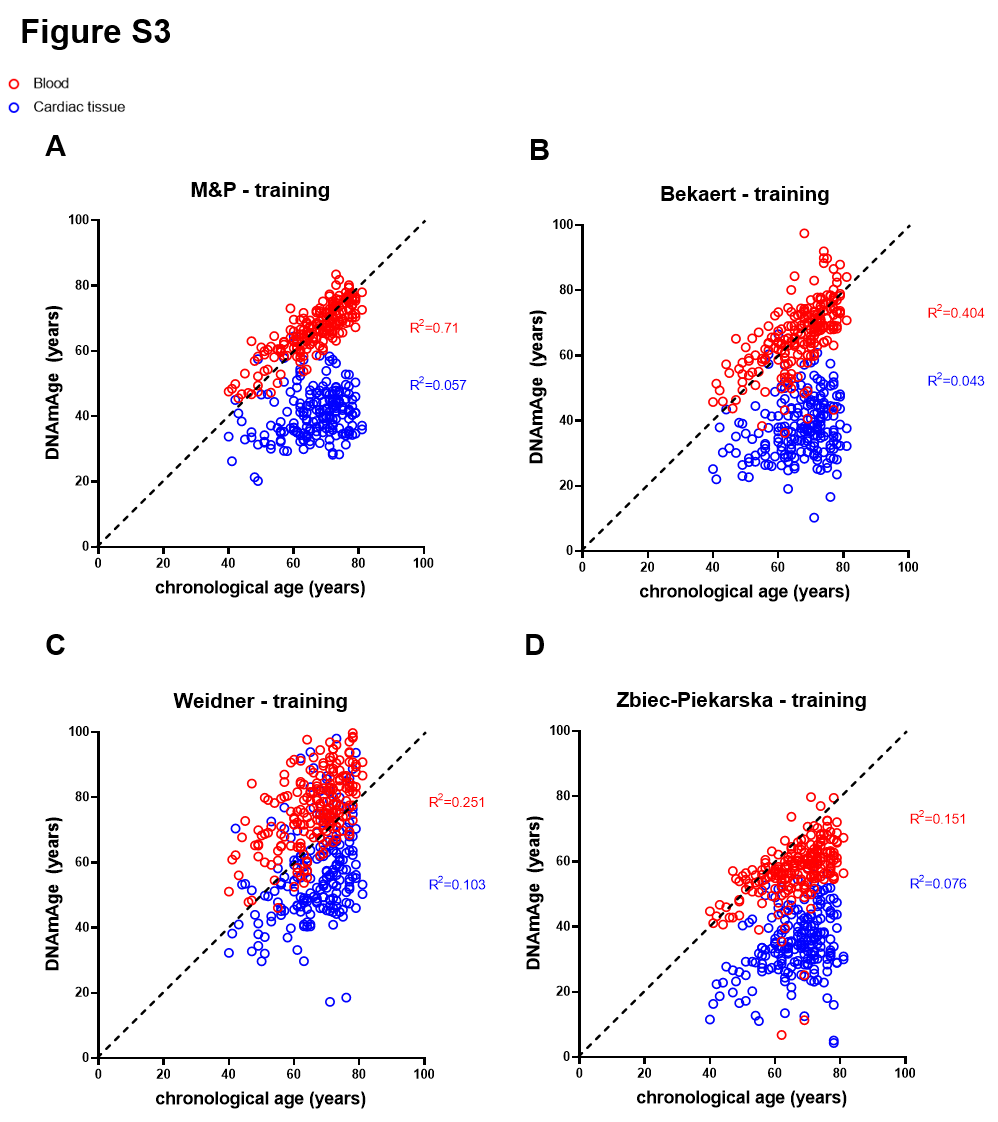

Supplement: Supplementary file 5 — Additional file 5. Figure S3. Blood models of epigenetic clocks in training group in blood and cardiac tissue samples. A M&P model; B Bekaert; C Weidner; D Zbiec-Piekarska. [file 13148_2023_1467_MOESM5_ESM.tif]

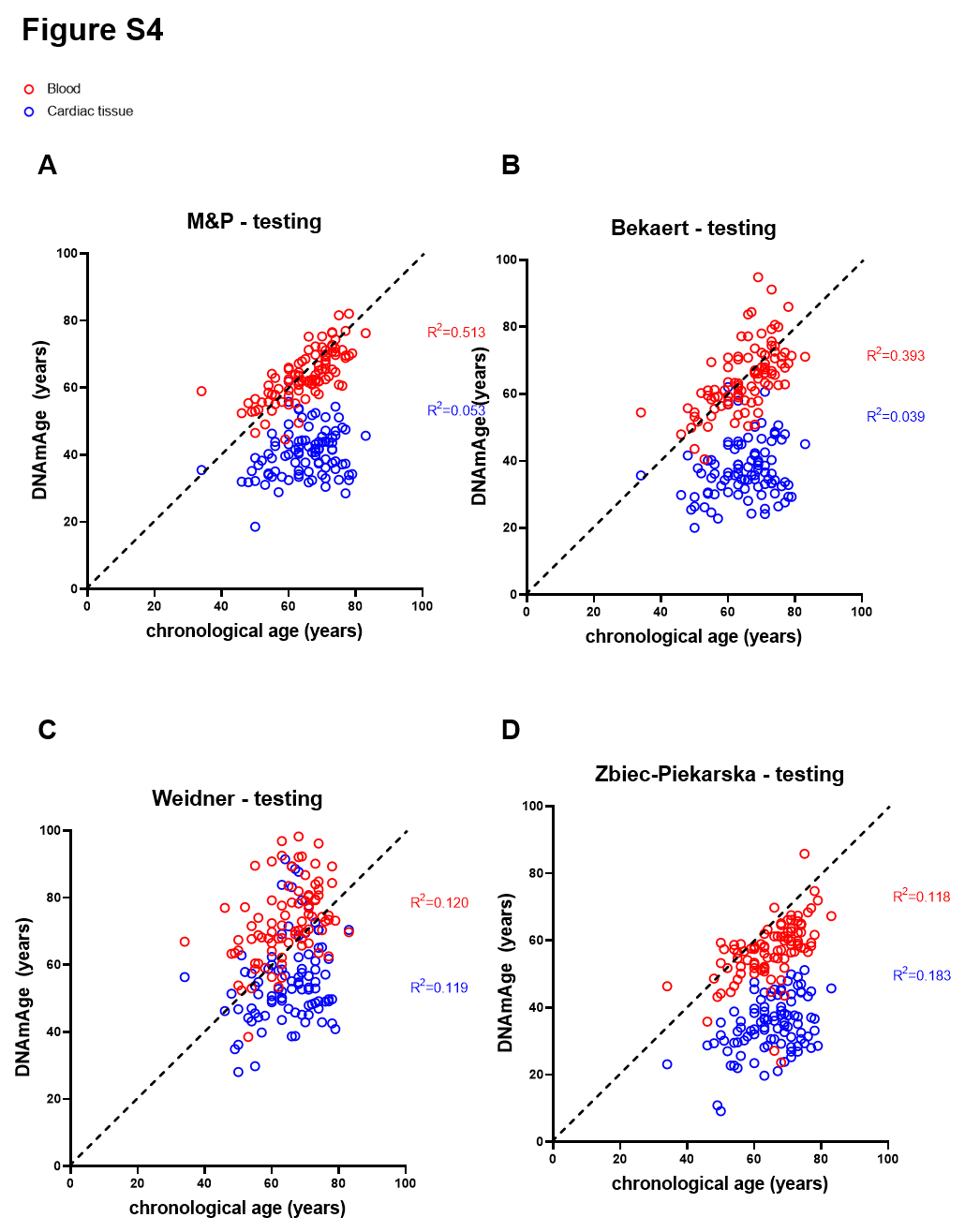

Supplement: Supplementary file 6 — Additional file 6. Figure S4. blood models of epigenetic clocks in the blood and cardiac tissue samples testing group. A M&P model; B Bekaert; C Weidner; D Zbiec-Piekarska. [file 13148_2023_1467_MOESM6_ESM.tif]

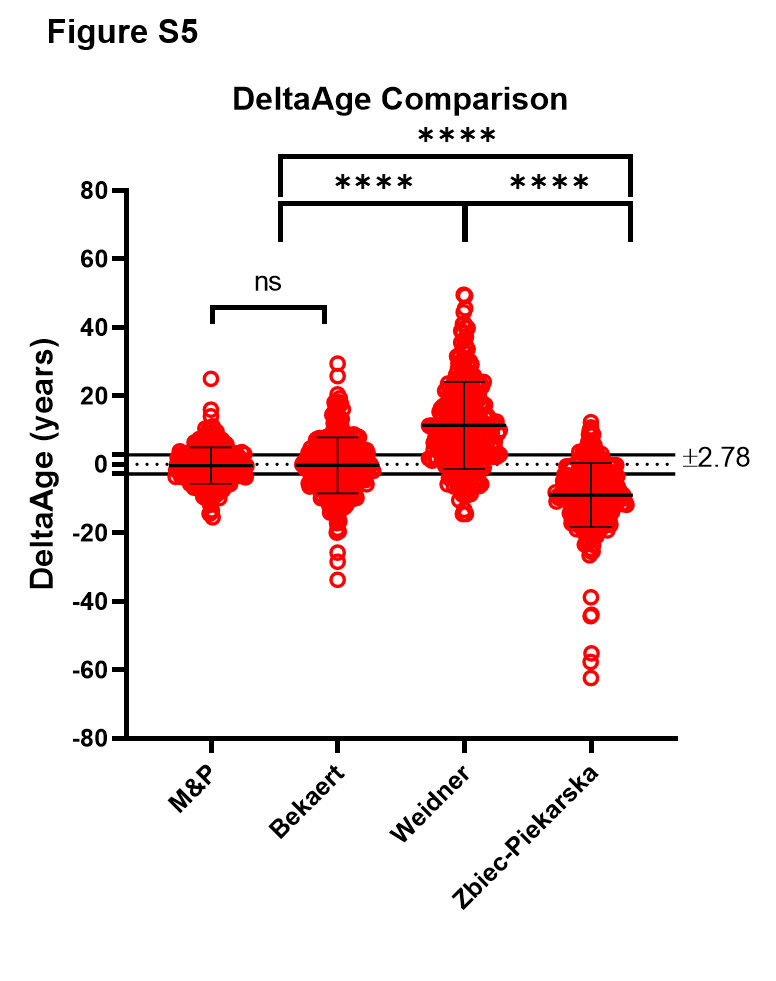

Supplement: Supplementary file 7 — Additional file 7. Figure S5. Comparison of blood DeltaAges. The value 2.78 refers to the MAD of the M&P blood-based epigenetic clock. [file 13148_2023_1467_MOESM7_ESM.tif]

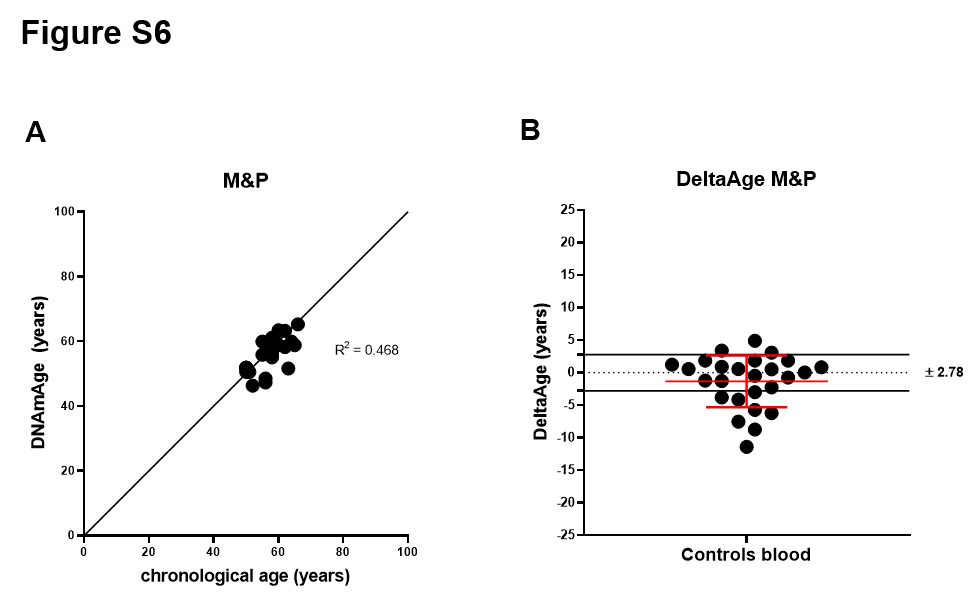

Supplement: Supplementary file 10 — Additional file 10. Figure S6. Blood of healthy control group (n = 26). A Correlation between chronological age (mean ± SD) 57.4 ± 4.8 and DNAmAge 56.1 ± 5.2 years estimated by M&P blood formula (paired t test > 0.05). B Blood DeltaAge of healthy controls mean ± SD (− 1.34 ± 4.0). 57.7% of DeltaAge falls within the range of normality (± 2.78); 30.8% of volunteers are decelerated, while 11.5% are accelerated. [file 13148_2023_1467_MOESM10_ESM.tif]

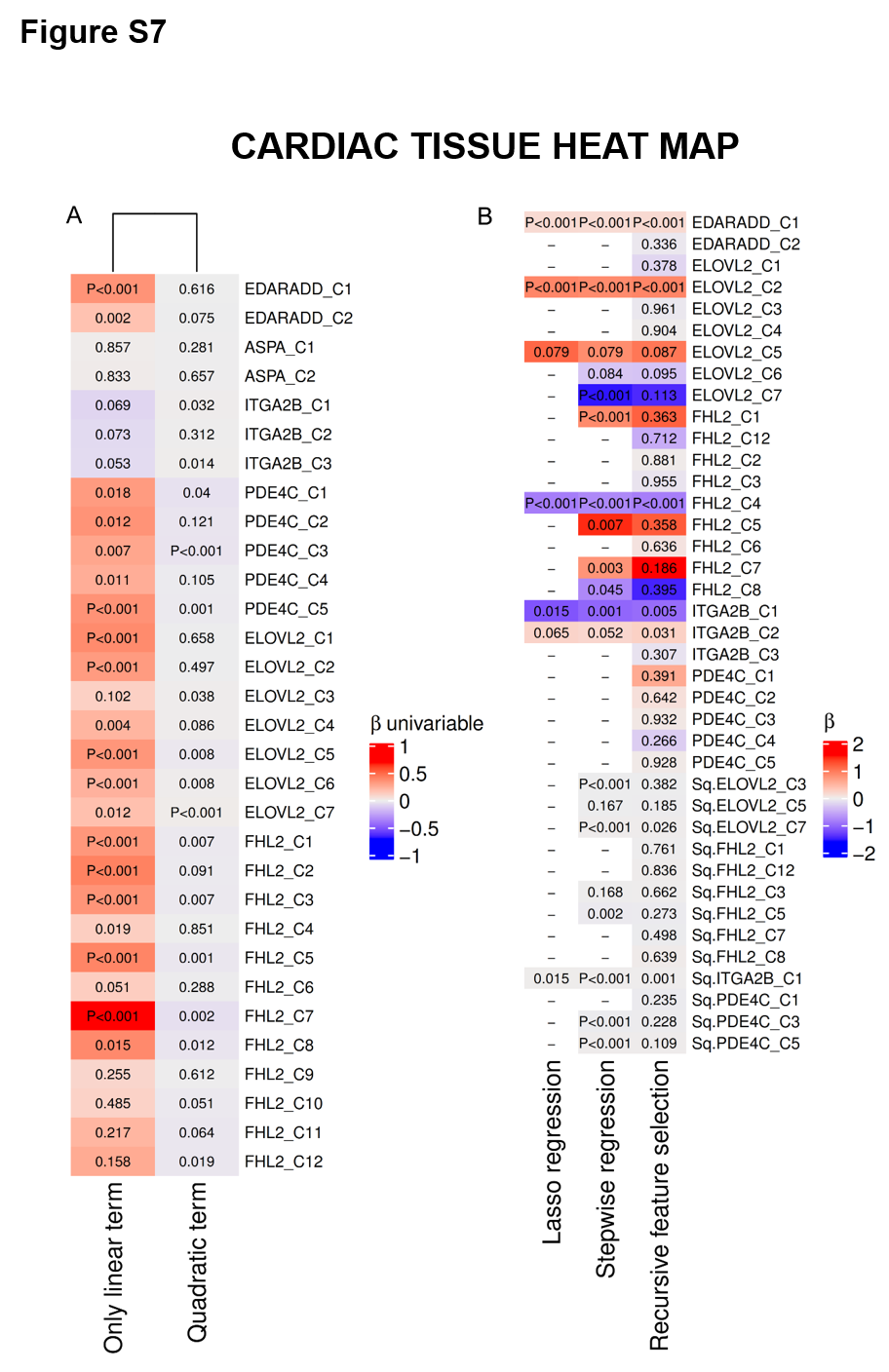

Supplement: Supplementary file 11 — Additional file 11. Figure S7. Heat map summarizing analyses of cardiac samples CpGs. A Results for all CpG analyzed in a univariable model. B Results from the Lasso regression, Stepwise regression, and Recursive feature selection for final model identification. Cell color represents the entity of the regression coefficient b for each CpG predicting the increase/decrease in aging in a univariable (A) and multivariable (B) analysis. Cell value represents the P value associated with the respective coefficients b. Variable name starting with “Sq.” indicates that the variable enters the model in the quadric form. [file 13148_2023_1467_MOESM11_ESM.tif]

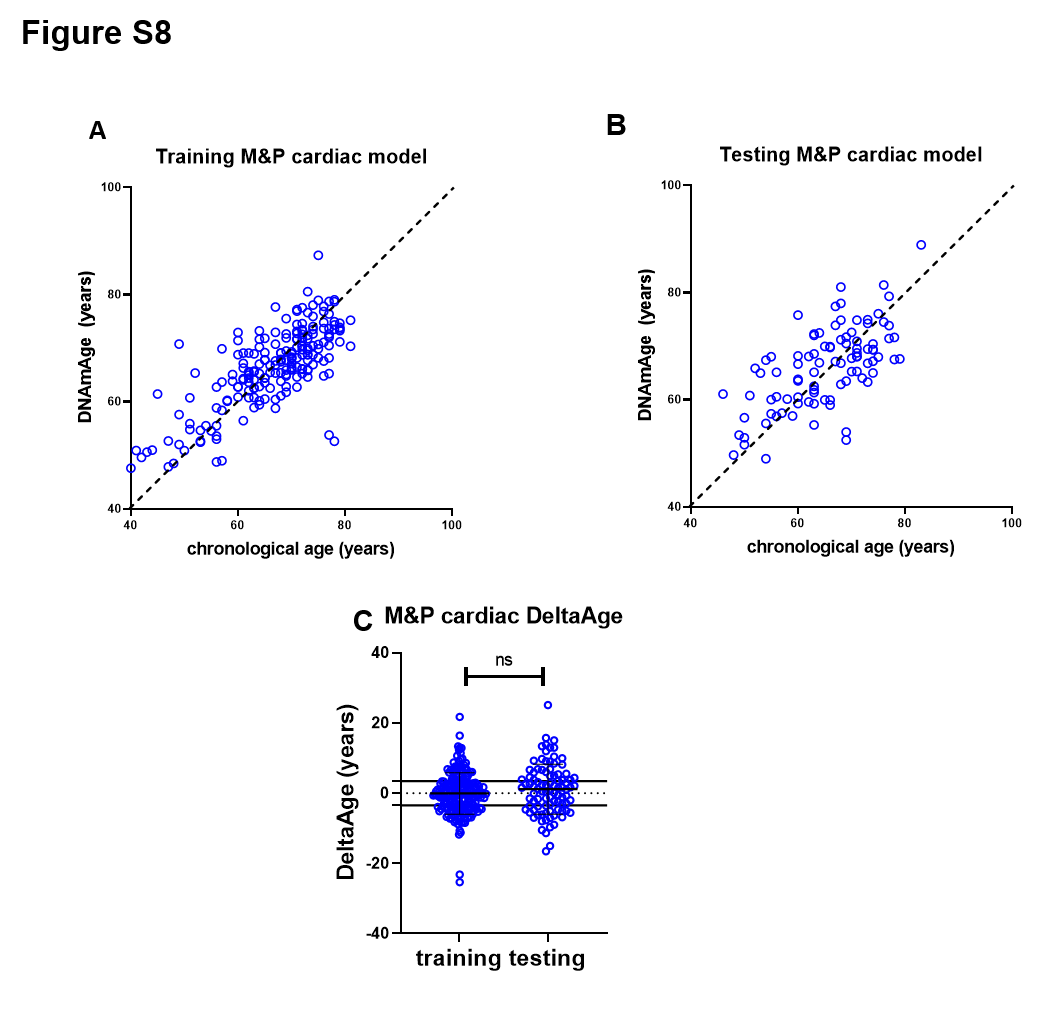

Supplement: Supplementary file 12 — Additional file 12. Figure S8. M&P cardiac model in training and testing groups. A Chronological versus DNAmAge in the training group; B Chronological versus DNAmAge in the testing group. C comparison of training and testing DeltaAges. No differences between cohorts (p value: ns). [file 13148_2023_1467_MOESM12_ESM.tif]

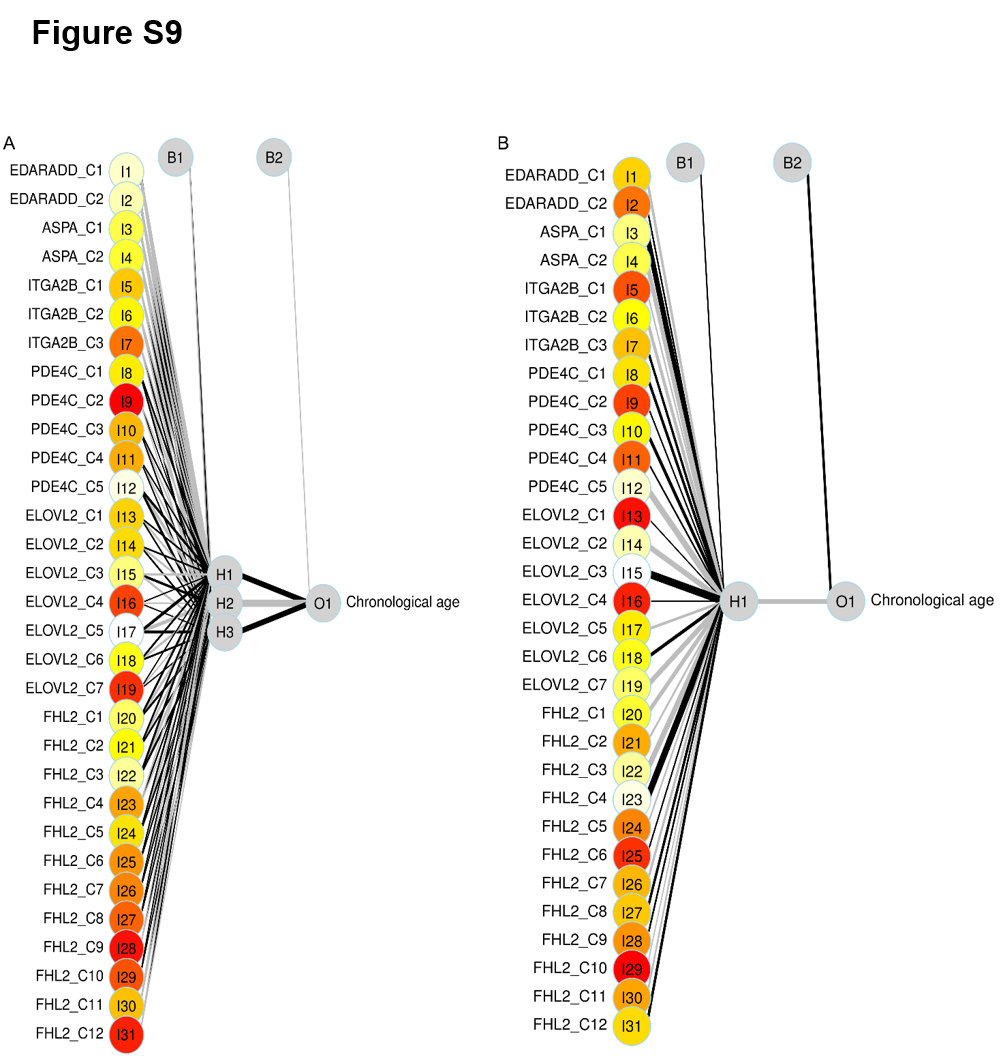

Supplement: Supplementary file 16 — Additional file 16. Figure S9. Diagrams of the Neural Networks employed for the blood and cardiac tissue. A Plot of the Neural Network for the chronological age prediction from blood CpGs samples. B Plot of the Neural Network for the chronological age prediction from cardiac CpGs samples. Each node represents an input CpG, while edges represent the weights between layers. The thickness of the edge is proportional to the magnitude of each weight. Positive weights are plotted as black lines; negative weights as grey lines. The Bias nodes cover the same role as an intercept in a regression model. I = input node, O =output node, H = hidden node, B = bias weight. [file 13148_2023_1467_MOESM16_ESM.tif]

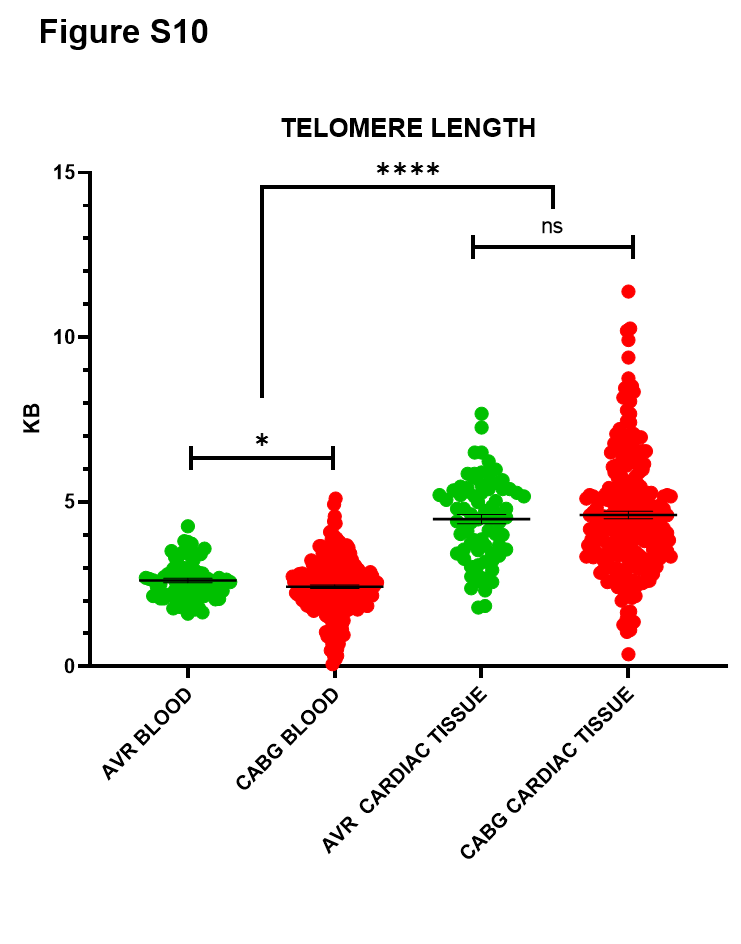

Supplement: Supplementary file 18 — Additional file 18. Figure S10. Telomere length (TL) of blood and cardiac tissue of AVR and CABG patients. The telomere length (kb) has been estimated by qPCR. Blood AVR TL mean and SD amount at 2.61 ± 0.57 kb; CABG blood TL is 2.42 ± 0.84 kb. Welch’s t test reveals a p value of 0.026. On the other hand, no differences arise in cardiac tissue (Welch’s t test p = 0.49) in which AVR TL is 4.48 kb ± 1.24 and CABG 4.60 ± 1.77 kb. [file 13148_2023_1467_MOESM18_ESM.tif]
