# Supplementary material for: Distinguishable DNA methylation defines a cardiac-specific epigenetic clock
Source: Clin Epigenetics. 2023 Mar 29;15:53. doi: 10.1186/s13148-023-01467-z (PMC10053964; doi:10.1186/s13148-023-01467-z)
Supplement: Supplementary file 2 — Additional file 2. Table S1. Correlation between chronological age and blood CpG Methylation levels in different datasets. Values in bold refer to significant correlations. [file 13148_2023_1467_MOESM2_ESM.docx]

| **Correlation between chronological age and DNA methylation in blood samples** | | | | | | | |
| --- | --- | --- | --- | --- | --- | --- | --- |
|  |  | Whole sample | | Training dataset | | Testing dataset | |
| **Gene** | CpG | r | R^2^ | r | R^2^ | r | R^2^ |
| **EDARADD** | C1 | **-0.405** | 0.164 | **-0.436** | 0.190 | **-0.330** | 0.109 |
|  | C2 | **-0.356** | 0.127 | **-0.419** | 0.176 | **-0.255** | 0.065 |
| **ASPA** | C1 | **-0.271** | 0.073 | **-0.270** | 0.073 | **-0.278** | 0.077 |
|  | C2 | **-0.283** | 0.080 | **-0.276** | 0.076 | **-0.280** | 0.078 |
| **ITGA2B** | C1 | **-0.321** | 0.103 | **-0.285** | 0.081 | **-0.404** | 0.163 |
|  | C2 | **-0.355** | 0.126 | **-0.340** | 0.116 | **-0.387** | 0.150 |
|  | C3 | **-0.304** | 0.092 | **-0.302** | 0.091 | **-0.296** | 0.088 |
| **PDE4C** | C1 | **0.202** | 0.041 | **0.237** | 0.056 | 0.160 | 0.026 |
|  | C2 | **0.205** | 0.042 | **0.204** | 0.042 | **0.229** | 0.052 |
|  | C3 | **0.207** | 0.043 | **0.270** | 0.0730 | 0.212 | 0.015 |
|  | C4 | **0.173** | 0.030 | **0.206** | 0.042 | 0.116 | 0.013 |
|  | C5 | **0.376** | 0.141 | **0.437** | 0.191 | **0.279** | 0.078 |
| **ELOVL2** | C1 | **0.422** | 0.178 | **0.406** | 0.165 | **0.474** | 0.225 |
|  | C2 | **0.366** | 0.134 | **0.328** | 0.108 | **0.455** | 0.207 |
|  | C3 | **0.118** | 0.014 | 0.060 | 0.004 | **0.280** | 0.078 |
|  | C4 | **0.200** | 0.040 | **0.137** | 0.019 | **0.371** | 0.138 |
|  | C5 | **0.548** | 0.300 | **0.551** | 0.304 | **0.546** | 0.298 |
|  | C6 | **0.419** | 0.176 | **0.386** | 0.149 | **0.530** | 0.281 |
|  | C7 | **0.328** | 0.108 | **0.300** | 0.090 | **0.397** | 0.158 |
| **FHL2** | C1 | **0.529** | 0.280 | **0.529** | 0.280 | **0.516** | 0.266 |
|  | C2 | **0.367** | 0.135 | **0.353** | 0.125 | **0.385** | 0.148 |
|  | C3 | **0.515** | 0.265 | **0.507** | 0.257 | **0.523** | 0.274 |
|  | C4 | **0.271** | 0.073 | **0.266** | 0.071 | **0.288** | 0.083 |
|  | C5 | **0.366** | 0.134 | **0.328** | 0.108 | **0.441** | 0.194 |
|  | C6 | **0.244** | 0.060 | **0.294** | 0.086 | 0.138 | 0.019 |
|  | C7 | **0.305** | 0.093 | **0.288** | 0.083 | **0.322** | 0.104 |
|  | C8 | **0.292** | 0.085 | **0.295** | 0.087 | **0.255** | 0.065 |
|  | C9 | **0.143** | 0.020 | **0.170** | 0.029 | 0.069 | 0.005 |
|  | C10 | 0.108 | 0.012 | **0.159** | 0.025 | -0.009 | 0.000 |
|  | C11 | 0.012 | 0.000 | 0.018 | 0.000 | -0.015 | 0.000 |
|  | C12 | 0.101 | 0.010 | 0.128 | 0.016 | 0.005 | 0.000 |
