# Supplementary material for: Distinguishable DNA methylation defines a cardiac-specific epigenetic clock
Source: Clin Epigenetics. 2023 Mar 29;15:53. doi: 10.1186/s13148-023-01467-z (PMC10053964; doi:10.1186/s13148-023-01467-z)
Supplement: Supplementary file 4 — Additional file 4. Table S2. Gene target and CpG were analyzed to estimate tissue-specific epigenetic clocks. [file 13148_2023_1467_MOESM4_ESM.docx]

| **Promoter gene target** | **CpG** | **hg38 position** | **Strand** | **ref** |
| --- | --- | --- | --- | --- |
| EDARADD | 1 | chr1:236.394.384 | - | Bekaert; M&P cardiac tissue |
|  | 2 | chr1:236.394.374 | - | M&P blood |
| ASPA | 1 | chr17:3.476.237 | + | M&P blood |
|  | 2 | chr17:3.476.273 | + | Weidner; Bekaert; |
| ITGA2B | 1 | chr17:44.390.375 | - | M&P cardiac tissue |
|  | 2 | chr17:44.390.361 | - | Weidner; M&P heart and cardiac tissue |
|  | 3 | chr17:44.390.359 | - |  |
| PDE4C | 1 | chr19:18.233.070 | + |  |
|  | 2 | chr19:18.233.079 | + |  |
|  | 3 | chr19:18.233.082 | + | M&P cardiac tissue |
|  | 4 | chr19:18.233.091 | + |  |
|  | 5 | chr19:18.233.104 | + | Weidner; Bekaert; M&P blood&cardiac tissue |
| ELOVL2 | 1 | chr6:11.044.428 | - |  |
|  | 2 | chr6:11.044.421 | - | M&P blood&cardiac tissue |
|  | 3 | chr6:11.044.416 | - | M&P blood |
|  | 4 | chr6:11.044.413 | - | M&P blood |
|  | 5 | chr6:11.044.411 | - | Zbiec-Piekarska1; M&P cardiac tissue |
|  | 6 | chr6:11.044.409 | - | Bekaert; M&P blood |
|  | 7 | chr6:11.044.406 | - | Zbiec-Piekarska1; M&P cardiac tissue |
| FHL2 | 1 | chr2:105.399.282 | + | Zbiec-Piekarska2; M&P cardiac tissue |
|  | 2 | chr2:105.399.288 | + | M&P blood |
|  | 3 | chr2:105.399.291 | + | M&P blood |
|  | 4 | chr2:105.399.297 | + | M&P blood& cardiac tissue |
|  | 5 | chr2:105.399.300 | + | M&P cardiac tissue |
|  | 6 | chr2:105.399.310 | + |  |
|  | 7 | chr2:105.399.314 | + | M&P cardiac tissue |
|  | 8 | chr2:105.399.316 | + | M&P cardiac tissue |
|  | 9 | chr2:105.399.323 | + |  |
|  | 10 | chr2:105.399.327 | + | M&P blood |
|  | 11 | chr2:105.399.338 | + |  |
|  | 12 | chr2:105.399.340 | + |  |
