# Supplementary material for: Distinguishable DNA methylation defines a cardiac-specific epigenetic clock
Source: Clin Epigenetics. 2023 Mar 29;15:53. doi: 10.1186/s13148-023-01467-z (PMC10053964; doi:10.1186/s13148-023-01467-z)
Supplement: Supplementary file 8 — Additional file 8. Table S3. Blood models comparison in training, testing, and whole sample groups. [file 13148_2023_1467_MOESM8_ESM.docx]

|  |  |  |  | corrected prediction | | |
| --- | --- | --- | --- | --- | --- | --- |
| **BLOOD** | **GROUP** | **MAD** | **SD** | **≤ 5.0 years** | **≤7.5 years** | **≤10.0 years** |
| **M&P** | **TRAINING** | **2.66** | **2.95** | **71.60** | **84.90** | **97.20** |
|  | **TESTING** | **3.09** | **3.88** | **60.00** | **82.10** | **93.70** |
|  | **TRAINING+TESTING** | **2.78** | **3.29** | **68.10** | **84.00** | **96.20** |
| **Bekaert** | **TRAINING** | **4.11** | **5.59** | **49.50** | **67.00** | **83.90** |
|  | **TESTING** | **5.58** | **5.60** | **49.50** | **65.30** | **80.00** |
|  | **TRAINING+TESTING** | **4.34** | **5.59** | **49.50** | **66.50** | **82.70** |
| **Weidner** | **TRAINING** | **9.59** | **11.08** | **24.50** | **37.70** | **47.70** |
|  | **TESTING** | **9.21** | **14.69** | **29.50** | **42.10** | **50.50** |
|  | **TRAINING+TESTING** | **9.55** | **12.27** | **26.00** | **39.00** | **48.60** |
| **Zbiec-Piekarska** | **TRAINING** | **7.29** | **8.68** | **29.90** | **40.70** | **58.80** |
|  | **TESTING** | **5.23** | **7.14** | **28.40** | **46.30** | **61.10** |
|  | **TRAINING+TESTING** | **7.08** | **8.24** | **29.40** | **42.40** | **59.50** |
