# Supplementary material for: Distinguishable DNA methylation defines a cardiac-specific epigenetic clock
Source: Clin Epigenetics. 2023 Mar 29;15:53. doi: 10.1186/s13148-023-01467-z (PMC10053964; doi:10.1186/s13148-023-01467-z)
Supplement: Supplementary file 9 — Additional file 9. Table S4. Correlation between epigenetic clocks and chronological age. The r and R2 of each epigenetic clock are reported. [file 13148_2023_1467_MOESM9_ESM.docx]

| Correlation between chronological age and model predicted age | | | | | | |
| --- | --- | --- | --- | --- | --- | --- |
|  | **Whole sample** | | **Training dataset** | | **Testing dataset** | |
| Model | **r** | **R^2^** | **r** | **R^2^** | **r** | **R^2^** |
| M&P cardiac specific | **0.721** | **0.520** | **0.765** | **0.585** | **0.633** | **0.401** |
| M&P blood | **0.808** | **0.653** | **0.842** | **0.709** | **0.729** | **0.531** |
| Bekaert | **0.636** | **0.404** | **0.636** | **0.404** | **0.627** | **0.393** |
| Weidner | **0.447** | **0.200** | **0.501** | **0.251** | **0.346** | **0.120** |
| Zbiec-Piekarska | **0.378** | **0.143** | **0.388** | **0.151** | **0.344** | **0.118** |
