# Supplementary material for: Distinguishable DNA methylation defines a cardiac-specific epigenetic clock
Source: Clin Epigenetics. 2023 Mar 29;15:53. doi: 10.1186/s13148-023-01467-z (PMC10053964; doi:10.1186/s13148-023-01467-z)
Supplement: Supplementary file 13 — Additional file 13. Table S5. Correlation between chronological age and cardiac tissue CpG Methylation levels in the different datasets. Values in bold refer to significant correlations. [file 13148_2023_1467_MOESM13_ESM.docx]

| Correlation between chronological age and DNA methylation in cardiac tissue samples | | | | | | | |
| --- | --- | --- | --- | --- | --- | --- | --- |
|  |  | **Whole sample** | | **Training dataset** | | **Testing dataset** | |
| Gene | **CpG** | **r** | **R^2^** | **r** | **R^2^** | **r** | **R^2^** |
| EDARADD | **C1** | **0.339** | **0.115** | **0.390** | **0.152** | **0.226** | **0.051** |
|  | **C2** | **0.196** | **0.038** | **0.203** | **0.041** | **0.181** | **0.033** |
| ASPA | **C1** | **-0.031** | **0.001** | **0.012** | **0.000** | **-0.132** | **0.017** |
|  | **C2** | **-0.035** | **0.001** | **0.014** | **0.000** | **-0.135** | **0.018** |
| ITGA2B | **C1** | **-0.106** | **0.011** | **-0.125** | **0.016** | **-0.047** | **0.002** |
|  | **C2** | **-0.103** | **0.011** | **-0.123** | **0.015** | **-0.031** | **0.001** |
|  | **C3** | **-0.114** | **0.013** | **-0.132** | **0.017** | **-0.045** | **0.002** |
| PDE4C | **C1** | **0.093** | **0.009** | **0.126** | **0.026** | **-0.069** | **0.005** |
|  | **C2** | **0.115** | **0.013** | **0.172** | **0.030** | **-0.015** | **0.000** |
|  | **C3** | **0.136** | **0.018** | **0.184** | **0.034** | **0.026** | **0.001** |
|  | **C4** | **0.123** | **0.015** | **0.174** | **0.030** | **-0.001** | **0.000** |
|  | **C5** | **0.261** | **0.068** | **0.328** | **0.108** | **0.115** | **0.013** |
| ELOVL2 | **C1** | **0.265** | **0.070** | **0.284** | **0.081** | **0.223** | **0.050** |
|  | **C2** | **0.279** | **0.078** | **0.262** | **0.069** | **0.323** | **0.104** |
|  | **C3** | **0.135** | **0.018** | **0.115** | **0.013** | **0.205** | **0.042** |
|  | **C4** | **0.231** | **0.053** | **0.203** | **0.041** | **0.340** | **0.116** |
|  | **C5** | **0.273** | **0.075** | **0.290** | **0.084** | **0.226** | **0.051** |
|  | **C6** | **0.261** | **0.068** | **0.244** | **0.060** | **0.314** | **0.099** |
|  | **C7** | **0.244** | **0.060** | **0.176** | **0.031** | **0.503** | **0.253** |
| FHL2 | **C1** | **0.294** | **0.086** | **0.295** | **0.087** | **0.283** | **0.080** |
|  | **C2** | **0.280** | **0.078** | **0.298** | **0.089** | **0.239** | **0.057** |
|  | **C3** | **0.276** | **0.076** | **0.291** | **0.085** | **0.231** | **0.053** |
|  | **C4** | **0.148** | **0.022** | **0.158** | **0.025** | **0.117** | **0.014** |
|  | **C5** | **0.325** | **0.106** | **0.324** | **0.105** | **0.324** | **0.105** |
|  | **C6** | **0.116** | **0.013** | **0.132** | **0.017** | **0.072** | **0.005** |
|  | **C7** | **0.321** | **0.103** | **0.343** | **0.118** | **0.206** | **0.068** |
|  | **C8** | **0.149** | **0.022** | **0.164** | **0.027** | **0.092** | **0.008** |
|  | **C9** | **0.065** | **0.004** | **0.077** | **0.006** | **0.020** | **0.000** |
|  | **C10** | **0.033** | **0.001** | **0.047** | **0.002** | **-0.046** | **0.002** |
|  | **C11** | **0.064** | **0.004** | **0.084** | **0.007** | **-0.010** | **0.000** |
|  | **C12** | **0.093** | **0.009** | **0.098** | **0.010** | **0.065** | **0.004** |
