# Supplementary material for: Distinguishable DNA methylation defines a cardiac-specific epigenetic clock
Source: Clin Epigenetics. 2023 Mar 29;15:53. doi: 10.1186/s13148-023-01467-z (PMC10053964; doi:10.1186/s13148-023-01467-z)
Supplement: Supplementary file 14 — Additional file 14. Table S6. The mean and median of different datasets in different epigenetic clocks. [file 13148_2023_1467_MOESM14_ESM.docx]

| Model | M&P cardiac specific | | M&P blood | | Bekaert | | Weidner | | Zbiec-Piekarska | |
| --- | --- | --- | --- | --- | --- | --- | --- | --- | --- | --- |
|  | **Mean** | **Median** | **Mean** | **Median** | **Mean** | **Median** | **Mean** | **Median** | **Mean** | **Median** |
| Whole samples | **4.73** | **3.81** | **4.17** | **3.42** | **6.44** | **5.04** | **13.36** | **10.38** | **9.95** | **8.77** |
| Training dataset | **4.29** | **3.57** | **3.83** | **3.08** | **6.46** | **5.03** | **13.03** | **10.60** | **10.22** | **8.87** |
| Testing dataset | **5.63** | **4.71** | **4.95** | **4.01** | **6.38** | **5.09** | **14.12** | **9.79** | **9.31** | **8.21** |
