# Supplementary material for: Distinguishable DNA methylation defines a cardiac-specific epigenetic clock
Source: Clin Epigenetics. 2023 Mar 29;15:53. doi: 10.1186/s13148-023-01467-z (PMC10053964; doi:10.1186/s13148-023-01467-z)
Supplement: Supplementary file 15 — Additional file 15. Table S7. Descriptive statistics of M&P cardiac model in training and testing groups. p value training versus testing: ns. [file 13148_2023_1467_MOESM15_ESM.docx]

|  |  |  |  | corrected prediction | | |
| --- | --- | --- | --- | --- | --- | --- |
|  | **GROUP** | **MAD** | **SD** | **≤ 5.0 years** | **≤7.5 years** | **≤10.0 years** |
| **Cardiac model** | **TRAINING** | **3.44** | **3.74** | **66.20** | **84.80** | **93.90** |
|  | **TESTING** | **3.64** | **4.47** | **53.20** | **74.50** | **86.20** |
|  | **TRAINING+TESTING** | **3.46** | **4.03** | **62.00** | **81.50** | **91.40** |
