# Supplementary material for: Distinguishable DNA methylation defines a cardiac-specific epigenetic clock
Source: Clin Epigenetics. 2023 Mar 29;15:53. doi: 10.1186/s13148-023-01467-z (PMC10053964; doi:10.1186/s13148-023-01467-z)
Supplement: Supplementary file 17 — Additional file 17. Table S8. Performance of the NNs for blood and cardiac tissue in training, testing, and whole sample groups. [file 13148_2023_1467_MOESM17_ESM.docx]

| NN Performance |  |  |  |  |  |
| --- | --- | --- | --- | --- | --- |
|  | **GROUP** | **Mean** | **SD** | **Median** | **MAD** |
| **BLOOD** | **TRAINING** | **5.04** | **3.66** | **4.28** | **3.80** |
|  | **TESTING** | **4.91** | **3.68** | **4.45** | **3.87** |
|  | **TRAINING+TESTING** | **5.00** | **3.66** | **4.35** | **3.83** |
| **CARDIO** | **TRAINING** | **3.88** | **3.01** | **3.44** | **3.02** |
|  | **TESTING** | **5.93** | **4.82** | **4.92** | **4.12** |
|  | **TRAINING+TESTING** | **4.55** | **3.82** | **3.74** | **3.28** |
