# Supplementary material for: Distinguishable DNA methylation defines a cardiac-specific epigenetic clock
Source: Clin Epigenetics. 2023 Mar 29;15:53. doi: 10.1186/s13148-023-01467-z (PMC10053964; doi:10.1186/s13148-023-01467-z)
Supplement: Supplementary file 19 — Additional file 19. Table S9. Sample size and percentage of DeltaAge distribution according to clinical classification. Differences between AVR and CABG have been tested utilizing Post Hoc Analysis for Pearson’s Chi-Squared Test. In blood, significant differences were found when comparing regular and accelerated groups; in the heart, the difference arises in the decelerated groups. Overall: Chi-Squared test. [file 13148_2023_1467_MOESM19_ESM.docx]

| Distribution | AVR (n=74) | | | CABG(n=239) | | | AVR vs CABG  (P-value) | | | (P-value) |
| --- | --- | --- | --- | --- | --- | --- | --- | --- | --- | --- |
|  | Decelerated | Normal | Accelerated | Decelerated | Normal | Accelerated | Decelerated | Normal | Accelerated | Overall |
| Blood | 29,7%  (n=22) | 56,8%  (n=42) | 13,5%  (n=10) | 33,0%  (n=79) | 36,5%  (n=87) | 30,5%  (n=73) | ns | 0,006 | 0,011 | 0.002 |
| **Distribution** | **AVR (n=68)** | | | **CABG(n=224)** | | | **AVR vs CABG**  **(P-value)** | | | **(P-value)** |
|  | Decelerated | Normal | Accelerated | Decelerated | Normal | Accelerated | Decelerated | Normal | Accelerated |  |
| Cardiac tissue | 39,7%  (n=27) | 36,8%  (n=25) | 23,5%  (n=16) | 24,5%  (n=55) | 48,2%  (n=108) | 27,2%  (n=61) | 0,045 | ns | ns | 0.049 |
